# Supplementary material for: Alkali metal reduction of alkali metal cations
Source: Nat Commun. 2023 Dec 9;14:8147. doi: 10.1038/s41467-023-43925-5 (PMC10709313; doi:10.1038/s41467-023-43925-5)
Supplement: Supplementary file 1 — Supplementary Information [file 41467_2023_43925_MOESM1_ESM.pdf]

Supplementary Information for

Alkali Metal Reduction of Alkali Metal Cations

Kyle G. Pearce, Han-Ying Liu, Samuel E. Neale, Hattie M. Goff, Mary F. Mahon, Claire L. McMullin* and Michael S. Hill*

Department of Chemistry, University of Bath, Claverton Down, Bath, BA2 7AY, United Kingdom

Synthetic Details

General Considerations 2

Synthesis of [{CH_2SiMe_2NDipp}$_2BeClNa$]$_2$ (7) 2

Synthesis of [{CH_2SiMe_2NDipp}$_2BeClK$]$_2$ (8) 3

Synthesis of [{CH_2SiMe_2NDipp}$_2BeClRb$]$_2$ (9) 3

Synthesis of [{CH_2SiMe_2NDipp}$_2BeClCs$]$_2$ (10) 3

Sequential Reduction Reactions from Compound 6 to 10 4

Attempted Reduction of Compound 10 to 9 4

Reduction of Compound 9 to 8 5

Attempted Reduction of Compound 8 to 7 5

NMR Spectra

Supplementary Figures 1-5: NMR Spectra for **[{CH_2SiMe_2NDipp}$_2BeClNa$]$_2$ (7)** 6

Supplementary Figures 6-10: NMR Spectra for **[{CH_2SiMe_2NDipp}$_2BeClK$]$_2$ (8)** 8

Supplementary Figures 11-15: NMR Spectra for **[{CH_2SiMe_2NDipp}$_2BeClRb$]$_2$ (9)** 11

Supplementary Figures 16-20: NMR Spectra for **[{CH_2SiMe_2NDipp}$_2BeClCs$]$_2$ (10)** 13

Supplementary Figures 21-22: NMR Spectra for the **Sequential Reduction Reactions** 16

Supplementary Figures 23-24: NMR Spectra for the **Attempted Reduction of 10 to 9** 17

Supplementary Figures 25-26: NMR Spectra for the **Reduction of 9 to 8** 18

Supplementary Figures 27-28: NMR Spectra for the **Attempted Reduction of 8 to 7** 19

Crystallographic Details 22

Computational Details 29

References 37

Synthetic Details

General Considerations

CAUTION: Beryllium and its compounds are extremely toxic. Suitable precautions (e.g., use of protective clothing, breathing apparatus, and a well-ventilated fume cupboard) should be taken for all manipulations involving these species.

All manipulations were carried out using standard Schlenk line and glovebox techniques under an inert atmosphere of argon. NMR experiments were conducted in J-Young tap NMR tubes prepared in a glovebox. NMR spectra were recorded on a Bruker BioSpin GmbH spectrometer operating at 400.13 MHz (^{1}H), 61.42 MHz (^{2}H), 100.62 MHz (^{13}C) and 56.2 MHz (^{9}Be) or on an Agilent ProPulse spectrometer operating at 194.3 MHz (^{7}Li). Elemental analyses were performed at Elemental Microanalysis Ltd., Okehampton, Devon, UK. Solvents were dried by passage through a commercially available solvent purification system and stored under argon in ampoules over 4 Å molecular sieves. C_6D_6, and C_6D_8 were purchased from Merck, dried over potassium before distilling and storage over molecular sieves. $\{CH_2SiMe_2N(H)Dipp\}_2$,[1] $[\{CH_2SiMe_2NDipp\}_2BeClLi]$,[2] and Na/NaCl were synthesised according to literature procedures.[3]

Synthesis of $[\{CH_2SiMe_2NDipp\}_2BeClNa]_2$ (7)

A toluene solution (*ca.* 20 cm^3) of $[\{CH_2SiMe_2NDipp\}_2BeClLi]_2$ (**6**, 100 mg, 0.18 mmol) was introduced to an ampoule with a pre-formed sodium mirror (4.1 mg, 0.18 mmol) and left to stir for 16 hours. The colourless solution was filtered away from the black powder and concentrated affording $[\{CH_2SiMe_2NDipp\}_2BeClNa]_2$ (**7**) as a colourless solid, though some decomposition was also observed. Yield: 80.2 mg, 79%.

^{1}H NMR (C_6D_6): δ = 7.11-7.02 (Ar-H, 6H), 3.86 (sept, C$\underline{H}$(CH$_3$)$_2$, $^3J_{HH}$ = 6.86 Hz, 4H), 1.29 (d, CH(C$\underline{H}_3$)$_2$, $^3J_{HH}$ = 6.88 Hz, 12H), 1.14 (s, SiCH$_2$, 4H), 0.96 (br d, CH(C$\underline{H}_3$)$_2$, $^3J_{HH}$ = 6.53 Hz, 12H), 0.21 (s, SiCH$_3$, 12H).

^{13}C{^{1}H} NMR (C_6D_6): δ = 154.1(*i*-C$_6$H$_3$), 147.6 (*o*-C$_6$H$_3$), 123.6 (*m*-C$_6$H$_3$), 121.9 (*p*-C$_6$H$_3$), 27.6 ($\underline{C}$H(CH$_3$)$_2$), 24.9 (CH($\underline{C}$H$_3$)$_2$), 24.5 (CH($\underline{C}$H$_3$)$_2$), 14.3 (SiCH$_2$), 0.7 (SiCH$_3$).

^{9}Be NMR (C_6D_6) δ = 9.1 (br s, $\omega_{1/2}$ = 435 Hz).

Anal. Calc. for $Be_1Cl_1Na_1Si_2N_2C_{30}H_{50}$: C, 64.07; H, 8.96; N, 4.98. Found: C, 64.34; H, 9.15; N, 4.76.

This product can alternatively also be prepared using 5 wt% Na/NaCl.

Synthesis of $[\{CH_2SiMe_2NDipp\}_2BeClK]_2$ (8)

$[\{CH_2SiMe_2NDipp\}_2BeClLi]_2$ (20 mg, 0.035 mmol) and KC_8 (**6**, 7.2 mg, 0.053 mmol) were introduced into a Young's NMR tube and C_6D_6 (0.6 cm^3) was added. The sample was sonicated for 10 minutes and inverted several times over 4 hours before the colourless solution was filtered away from the suspension and concentrated affording a colourless solid. Colourless crystals of $[\{CH_2SiMe_2NDipp\}_2BeClK]_2$ (**8**) were grown from a saturated benzene solution and isolated. Yield: 11 mg, 54%.

^{1}H NMR (C_6D_6): δ = 7.11-6.72 (Ar-H, 6H), 3.93 (sept, C$\underline{H}$(CH_3)$_2$, $^3J_{HH}$ = 6.84 Hz, 4H), 1.31 (d, CH(C$\underline{H}_3$)$_2$, $^3J_{HH}$ = 6.92 Hz, 12H), 1.14 (s, $SiCH_2$, 4H), 0.95 (d, CH(C$\underline{H}_3$)$_2$, $^3J_{HH}$ = 6.99 Hz, 12H), 0.19 (s, $SiCH_3$, 12H).

^{13}C{^{1}H} NMR (C_6D_6): δ = 155.2 (*i*-C_6H_3), 147.9 (*o*-C_6H_3), 122.3 (*m*-C_6H_3), 120.6 (*p*-C_6H_3), 27.7 ($\underline{C}$H(CH_3)$_2$), 24.7 (CH($\underline{C}H_3$)$_2$), 24.3 (CH($\underline{C}H_3$)$_2$), 14.5 ($SiCH_2$), 0.7 ($SiCH_3$).

^{9}Be NMR (C_6D_6) δ = 9.8 (br s, $\omega_{1/2}$ = 399 Hz).

Anal. Calc. for $Be_1Cl_1Na_1Si_2N_2C_{30}H_{50}$: C, 62.29; H, 8.71; N, 4.84. Found: C, 63.19; H, 5.31; N, 3.95.

This reaction can also be performed using a K mirror.

Synthesis of $[\{CH_2SiMe_2NDipp\}_2BeClRb]_2$ (9)

Rb metal was added to a toluene solution of $[\{CH_2SiMe_2NDipp\}_2BeClLi]_2$ (**6**, 20 mg, 0.035 mmol) and sonicated for 15 minutes. The colourless solution was filtered away from the suspension and concentrated affording $[\{CH_2SiMe_2NDipp\}_2BeClRb]_2$ (**9**) as a colourless solid. Yield: 16 mg, 73%.

^{1}H NMR (C_6D_8): δ = 6.79 (m, *o*-Ar-H, 4H), 6.70 (br t, *p*-ArH, $^3J_{HH}$ = 6.99 Hz, 2H), 3.95 (sept, C$\underline{H}$(CH_3)$_2$, $^3J_{HH}$ = 6.77 Hz, 4H), 1.29 (d, CH(C$\underline{H}_3$)$_2$, $^3J_{HH}$ = 6.94 Hz, 12H), 1.08 (s, $SiCH_2$, 4H), 0.96 (d, CH(C$\underline{H}_3$)$_2$, $^3J_{HH}$ = 6.94 Hz, 12H), 0.12 (s, $SiCH_3$, 12H).

^{13}C{^{1}H} NMR (C_6D_8): δ = 155.3 (*i*-C_6H_3), 147.7 (*o*-C_6H_3), 122.6 (*m*-C_6H_3), 120.4 (*p*-C_6H_3), 27.6 ($\underline{C}$H(CH_3)$_2$), 24.7 (CH($\underline{C}H_3$)$_2$), 24.2 (CH($\underline{C}H_3$)$_2$), 14.6 ($SiCH_2$), 0.6 ($SiCH_3$).

^{9}Be NMR (C_6D_8) δ = 11.1 (br s, $\omega_{1/2}$ = 399 Hz).

Anal. Calc. for $Be_1Cl_1Rb_1Si_2N_2C_{30}H_{50}$: C, 57.67; H, 8.07; N, 4.48. Found: C, 58.78; H, 8.62; N, 4.26.

Synthesis of $[\{CH_2SiMe_2NDipp\}_2BeClCs]_2$ (10)

Cs metal was added to the top of a Young's NMR tube containing $[\{CH_2SiMe_2NDipp\}_2BeClLi]_2$ (**6**, 10 mg, 0.018 mmol) in C_6D_6 (0.6 cm^3). Upon melting the Cs (by hand) an orange colouration and black

precipitate was instantly observed and the reaction mixture was immediately filtered into a vial and placed on-top of the glovebox freezer, affording a small number of colourless crystals of $[\{CH_2SiMe_2NDipp\}_2BeClCs]_2$ (**10**) after 7 days. This compound was found to be unstable towards vacuum.

^{1}H NMR (C_6D_6): δ = 6.81 (d, *o*-Ar-H, $^3J_{HH}$ = 7.54 Hz, 4H), 6.66 (t, *p*-Ar-H, $^3J_{HH}$ = 7.59 Hz, 2H), 4.04 (sept, C$\underline{H}$(CH_3)$_2$, $^3J_{HH}$ = 6.88 Hz, 4H), 1.34 (d, CH(C$\underline{H}_3$)$_2$, $^3J_{HH}$ = 7.01 Hz, 12H), 1.17 (s, $SiCH_2$, 4H), 1.05 (d, CH(C$\underline{H}_3$)$_2$, $^3J_{HH}$ = 6.89 Hz, 12H), 0.2 (s, $SiCH_3$, 12H).

^{13}C{^{1}H} NMR (C_6D_6): δ = 155.4 (*i*-C_6H_3), 147.6 (*m*-C_6H_3), 123.0 (*o*-C_6H_3), 120.6 (*p*-C_6H_3), 27.7 ($\underline{C}$H(CH_3)$_2$), 25.1 (CH($\underline{C}H_3$)$_2$), 24.3 (CH($\underline{C}H_3$)$_2$), 14.7 ($SiCH_2$), 0.8 ($SiCH_3$).

^{9}Be NMR (C_6D_6) δ = 11.3 (br s, $\omega_{1/2}$ = 392 Hz).

Sequential Reduction Reactions from Compound 6 to 10

A C_6D_6 solution of $[\{CH_2SiMe_2NDipp\}_2BeClLi]_2$ (**6**; 4 mg, 0.007 mmol) was added to an ampoule and vigorously stirred over a pre-formed sodium mirror (0.4 mg, 0.017 mmol) for 8 hours before being filtered into a Young's NMR tube, observing the formation of compound **7**. The solution was then introduced to an ampoule and vigorously stirred over a pre-formed K mirror (6 mg, 0.15 mmol) for 8 hours before being filtered into a fresh Young's NMR tube, affording compound **8**. The solution of **8** was then placed into a Young's NMR tube containing Rb metal and sonicated for 20 minutes, exclusively affording compound **9**. The solution of **9** was then filtered into a vial containing Cs metal, agitated for 1 minute and immediately filtered into a Young's NMR tube observing the formation of compound **10**.

The transformation of compound **7** to **8** can also be performed using KC_8, though this could potentially necessitate a cation exchange rather than mutual alkali metal reduction.

Attempted Reduction of Compound 10 to 9

$[\{CH_2SiMe_2NDipp\}_2BeClCs]_2$ (**10**) in C_6D_6 (0.6 cm^3) was introduced to a Young's NMR tube containing Rb metal. The reaction mixture was sonicated for 20 minutes but only the observation of **10** was observed, even after sonicating for 1 hour, only compound **10** was observed.

Reduction of Compound 9 to 8

[{CH_2SiMe_2NDipp}$_2$BeClRb]$_2$ (**9**; 5 mg, 0.004 mmol) in C_6D_6 was introduced into an ampoule and vigorously stirred over a pre-formed K-mirror (6 mg, 0.15 mmol) for 4 hours before being filtered into a Young's NMR tube, observing the formation of compound **8**.

The transformation of compound **9** to **8** can also be performed *via* a cation exchange reaction with KC_8.

Attempted Reduction of Compound 8 to 7

[{CH_2SiMe_2NDipp}$_2$BeClK]$_2$ (**8**; 5 mg, 0.004 mmol) in C_6D_6 was introduced into an ampoule and vigorously stirred over a pre-formed Na-mirror (2 mg, 0.09 mmol) for 4 hours before being filtered into a Young's NMR tube. No evidence of reaction could be observed.

This reaction was also performed with 5 wt% Na/NaCl. Again, only the continued integrity **8** could be observed.

NMR Spectra

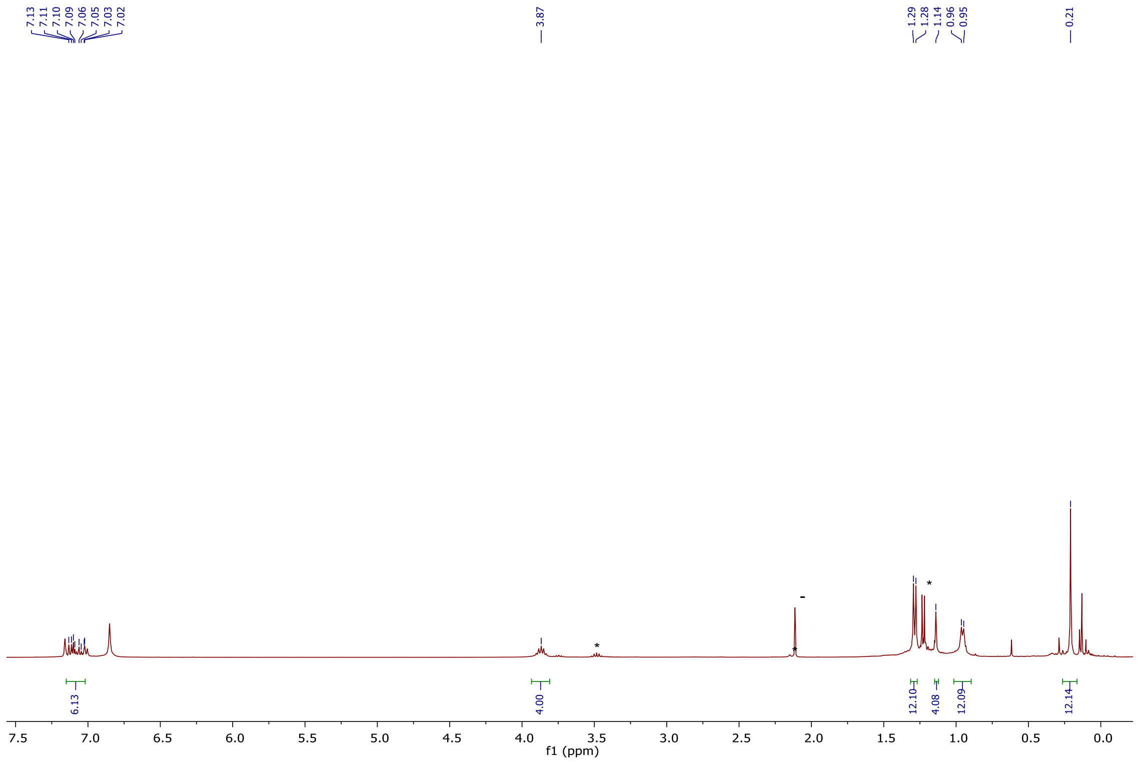

Supplementary Figure 1. ^{1}H NMR Spectrum (C_6D_6, 298 K, 400.13 MHz) for **[{CH$_2$SiMe$_2$NDipp}$_2$BeClNa]$_2$ (7).** * = [{CH$_2$SiMe$_2$NDipp}H]$_2$, - = residual toluene.

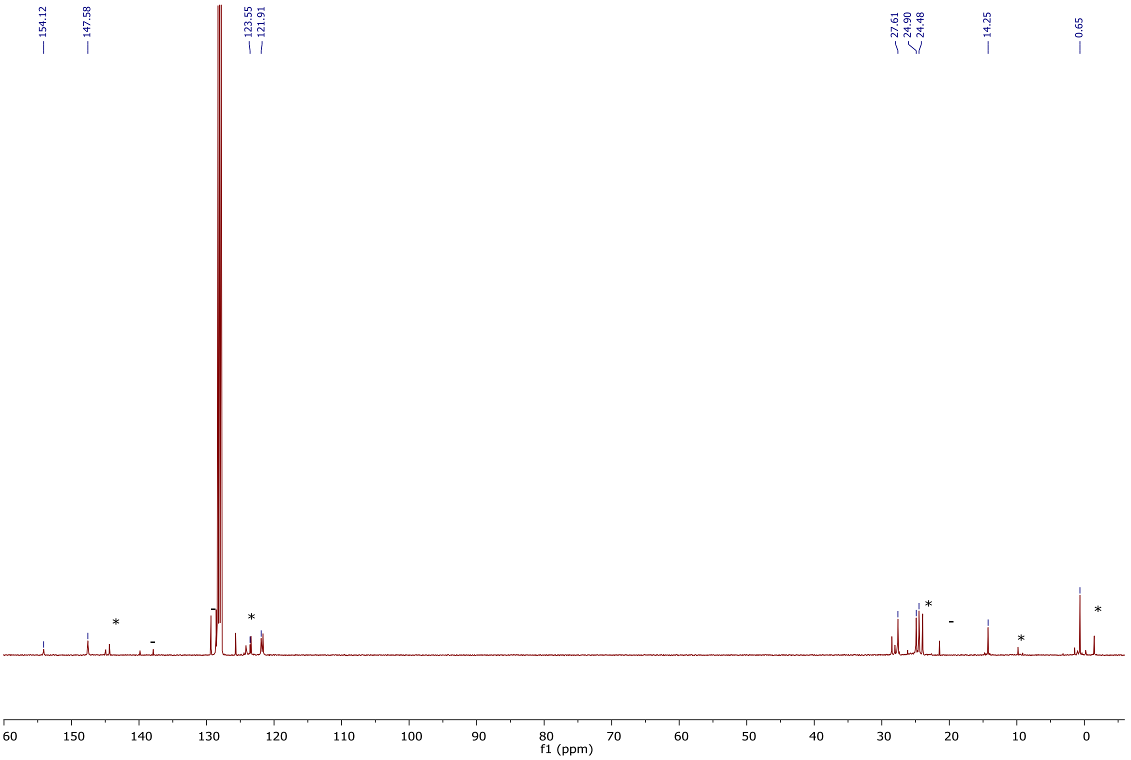

Supplementary Figure 2. $^{13}C\{^{1}H\}$ NMR Spectrum (C_6D_6, 298 K, 100.62 MHz) for **[{CH$_2$SiMe$_2$NDipp}$_2$BeClNa]$_2$ (7).** * = [{CH$_2$SiMe$_2$NDipp}H]$_2$, - = residual toluene.

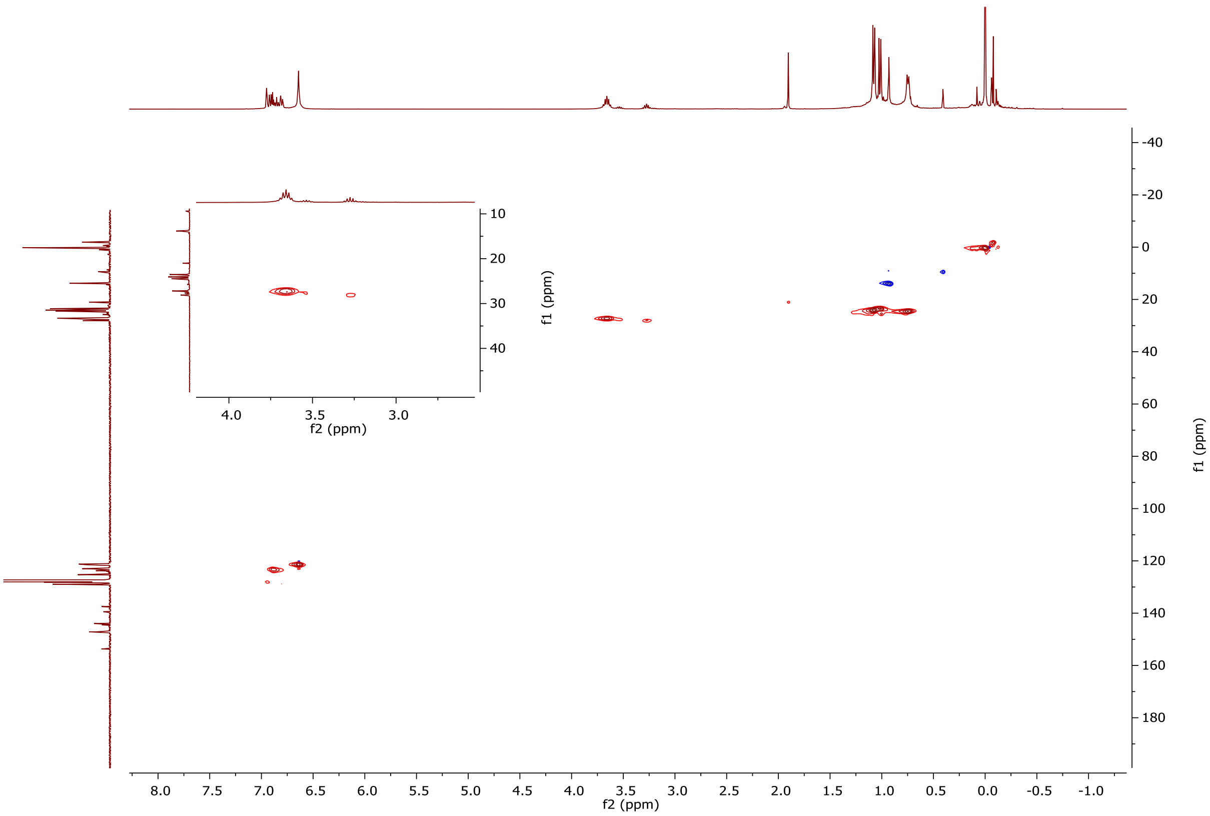

Supplementary Figure 3. ^{1}H-^{13}C HSQC trace (C_6D_6, 298 K, 400.13, 100.62 MHz) for **[{CH_2SiMe_2NDipp}$_2$$BeClNa$]$_2$ (7).**

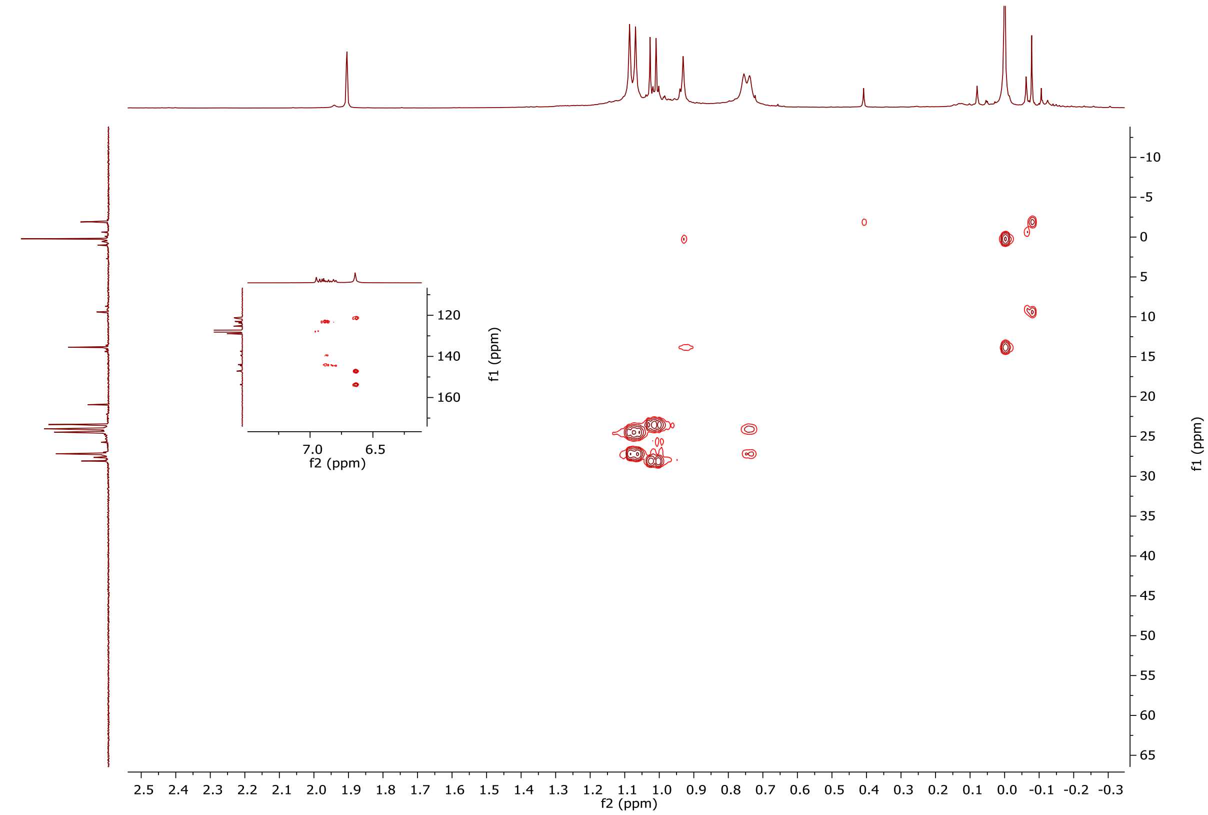

Supplementary Figure 4. ^{1}H-^{13}C HMBC trace (C_6D_6, 298 K, 400.13, 100.62 MHz) for **[{CH_2SiMe_2NDipp}$_2$$BeClNa$]$_2$ (7).**

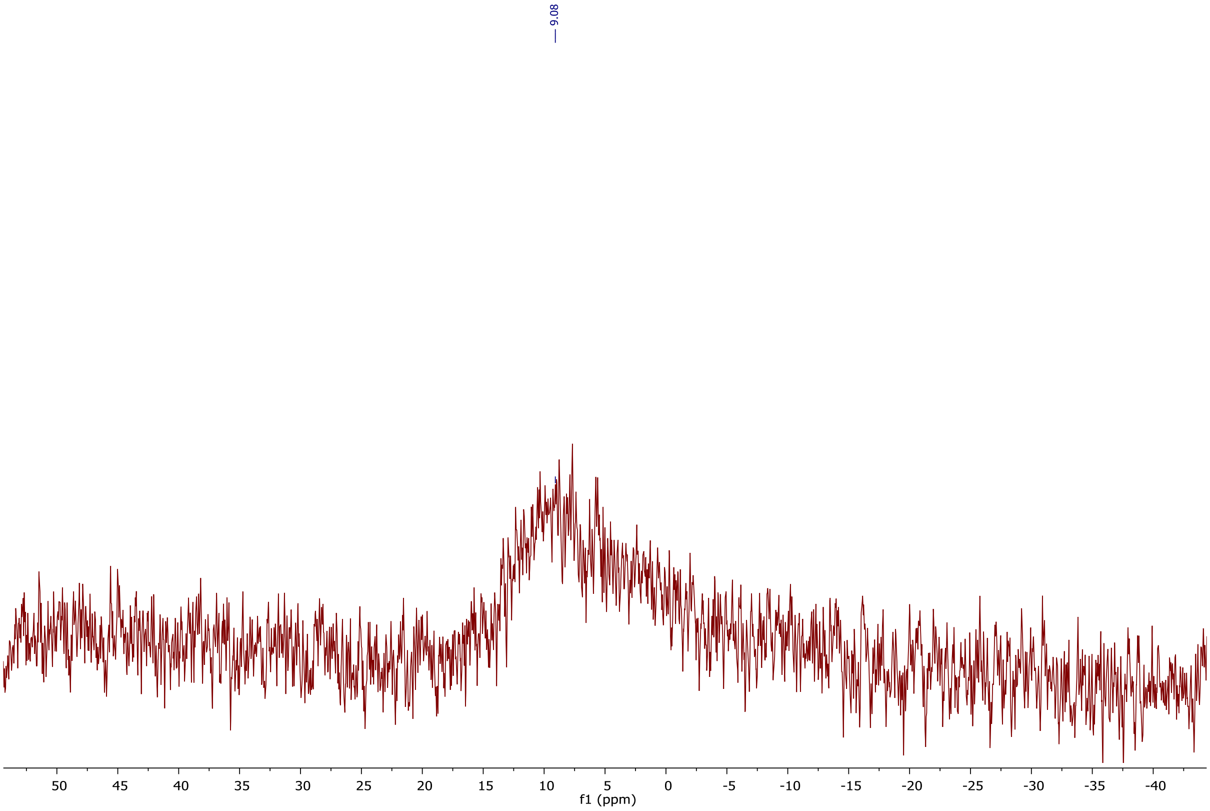

Supplementary Figure 5. ^{9}Be NMR Spectrum (C_6D_6, 298 K, 56.2 MHz) for **[{CH$_2$SiMe$_2$NDipp}$_2$BeClNa]$_2$ (7).**

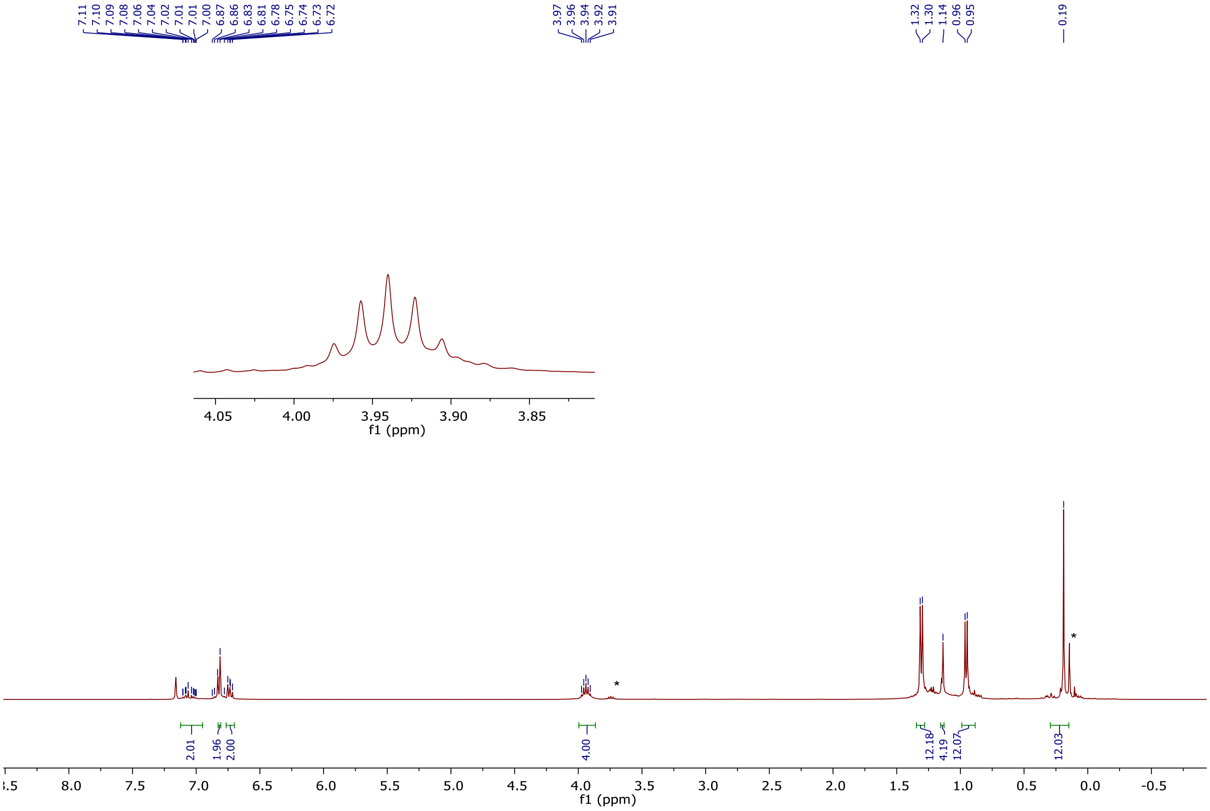

Supplementary Figure 6. ^{1}H NMR Spectrum (C_6D_6, 298 K, 400.13 MHz) for **[{CH$_2$SiMe$_2$NDipp}$_2$BeClK]$_2$** (**8**).* = **{CH$_2$SiMe$_2$NDipp}$_2$Be**.

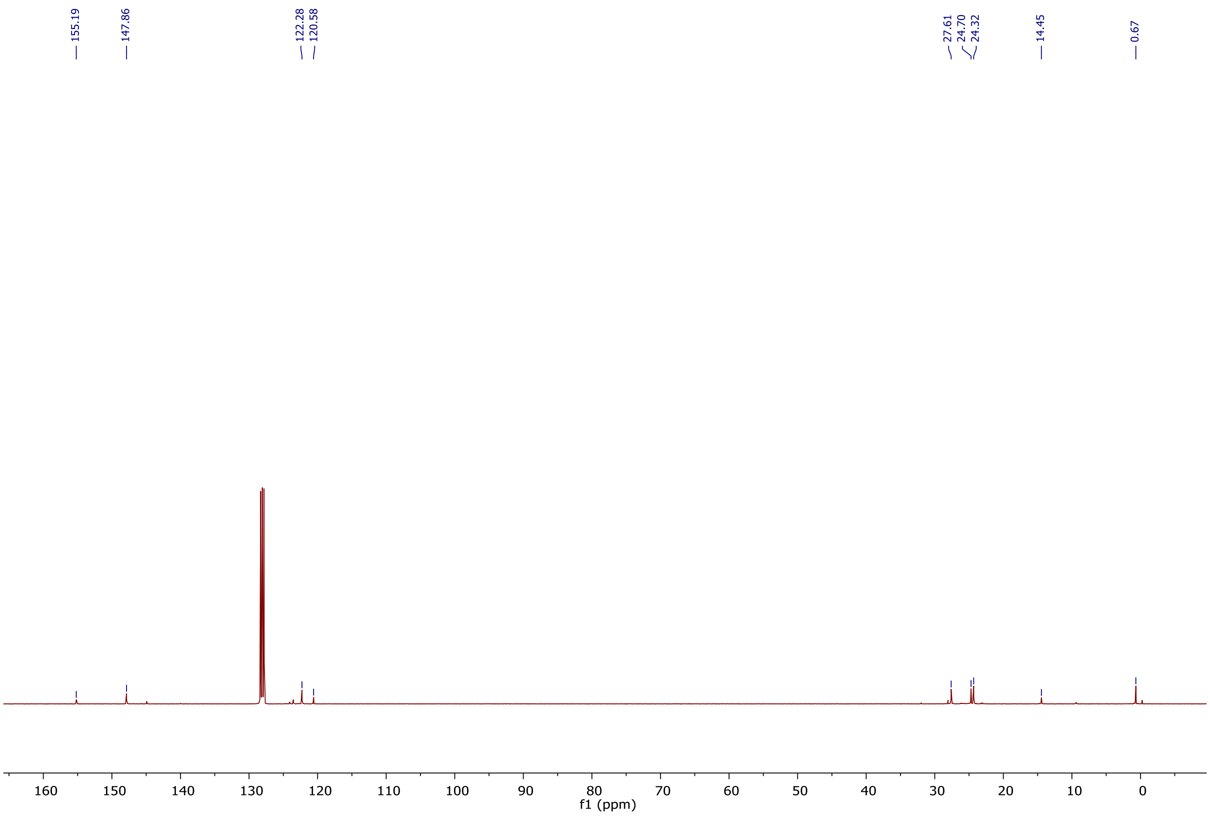

Supplementary Figure 7. $^{13}C\{^{1}H\}$ NMR Spectrum (C_6D_6, 298 K, 100.62 MHz) for **[{CH$_2$SiMe$_2$NDipp}$_2$BeClK]$_2$ (8).**

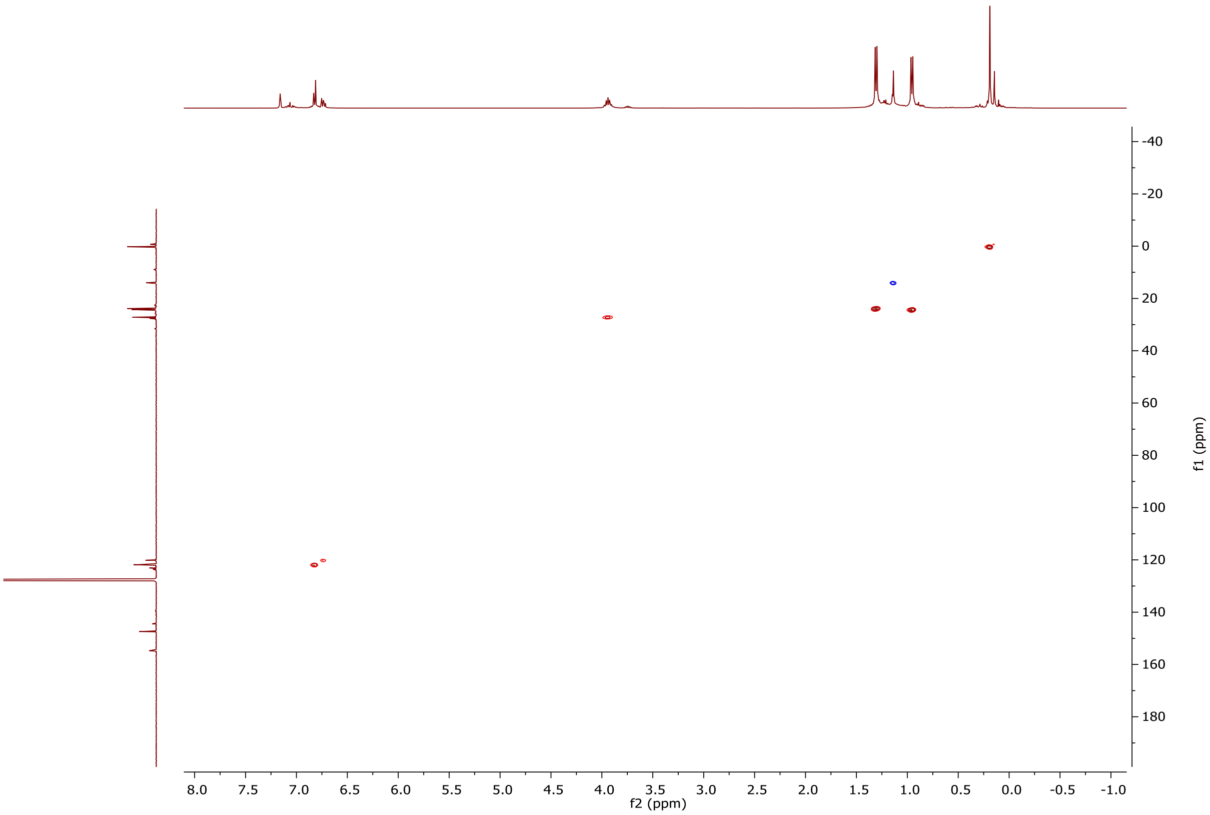

Supplementary Figure 8. ^{1}H-^{13}C HSQC trace (C_6D_6, 298 K, 400.13, 100.62 MHz) for **[{CH$_2$SiMe$_2$NDipp}$_2$BeClK]$_2$ (8).**

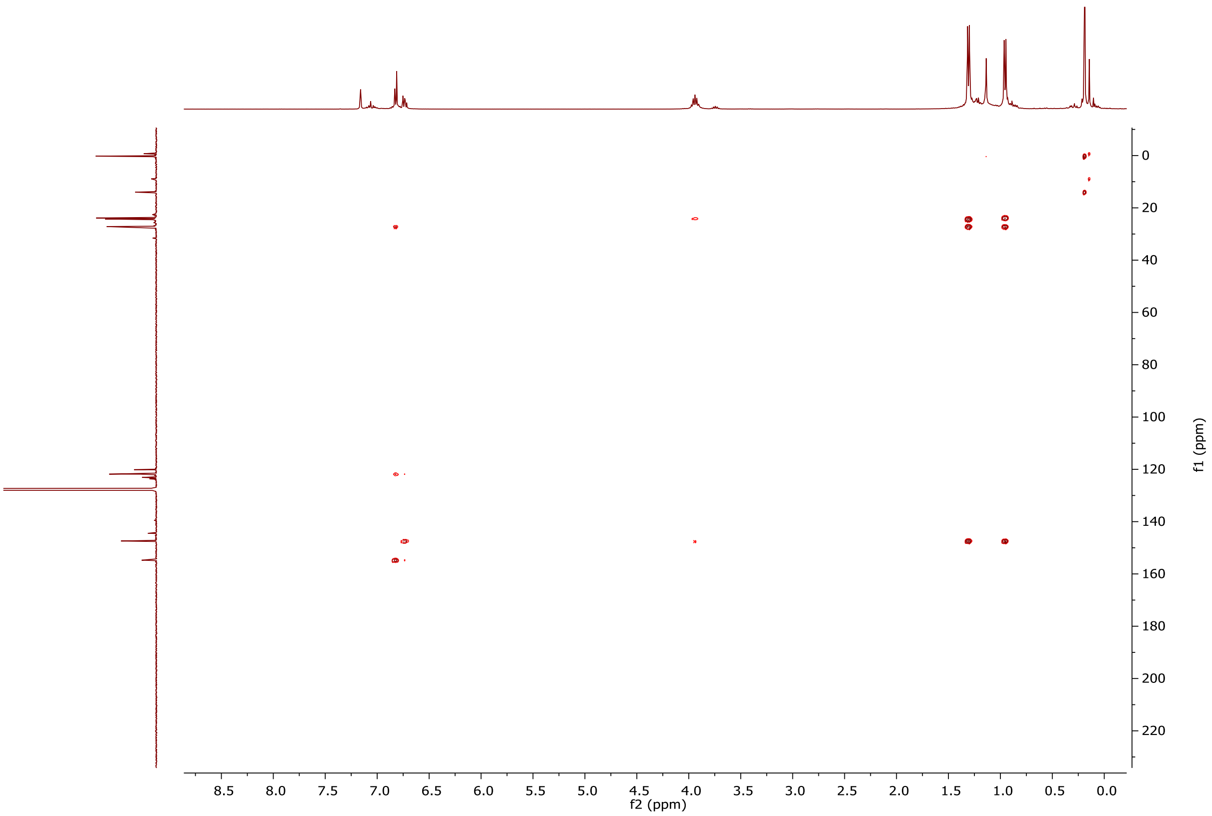

Supplementary Figure 9. ^{1}H-^{13}C HMBC trace (C_6D_6, 298 K, 400.13, 100.62 MHz) for **[{CH_2SiMe_2NDipp}$_2BeClK$]$_2$ (8).**

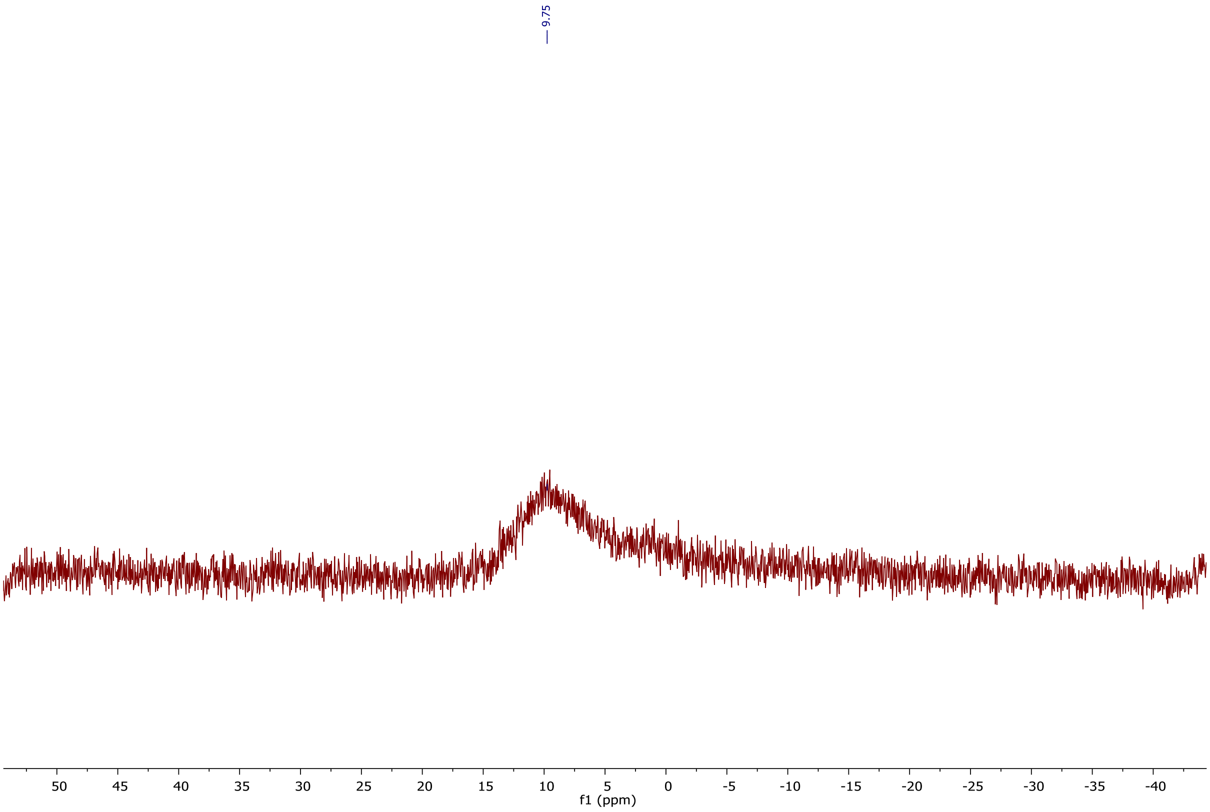

Supplementary Figure 10. [9]Be NMR Spectrum (C_6D_6, 298 K, 56.2 MHz) for **[{CH_2SiMe_2NDipp}$_2BeClK$]$_2$ (8).**

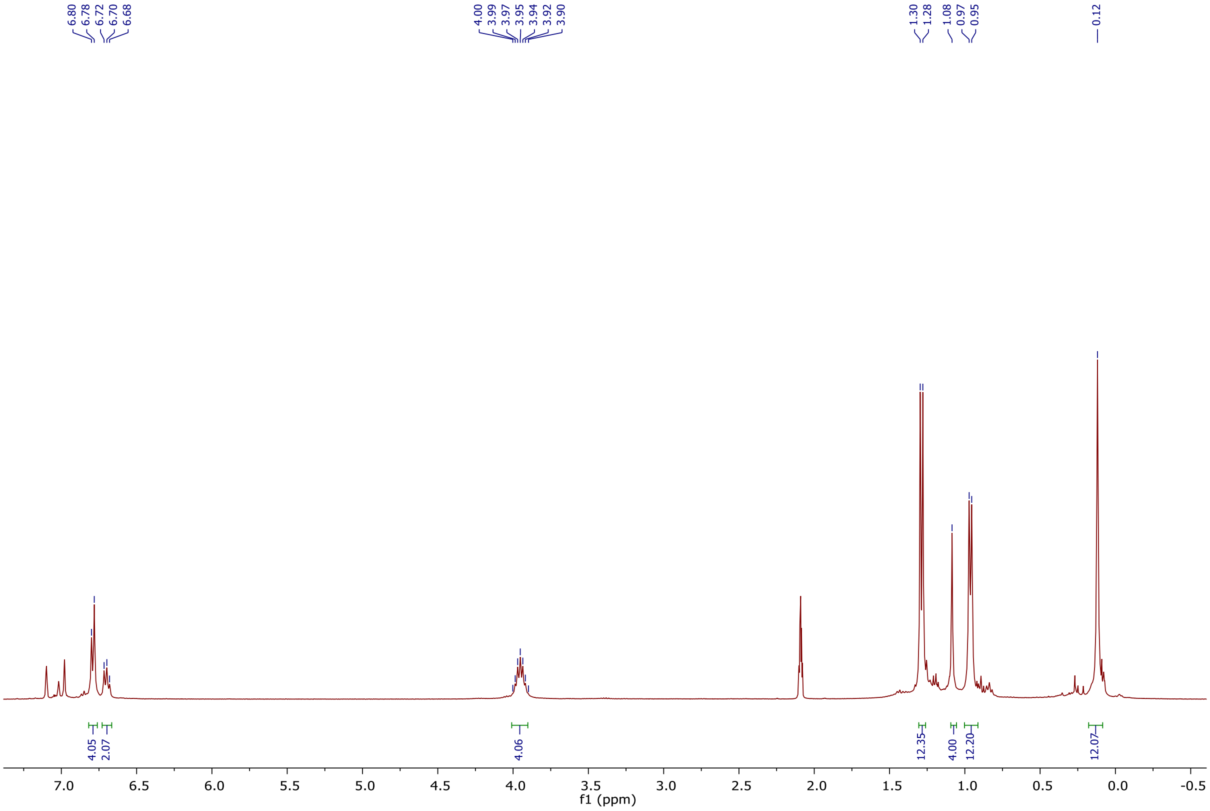

Supplementary Figure 11. ^{1}H NMR Spectrum (C_6D_8, 298 K, 400.13 MHz) for **$[\{CH_2SiMe_2NDipp\}_2BeClRb]_2$** (**9**).

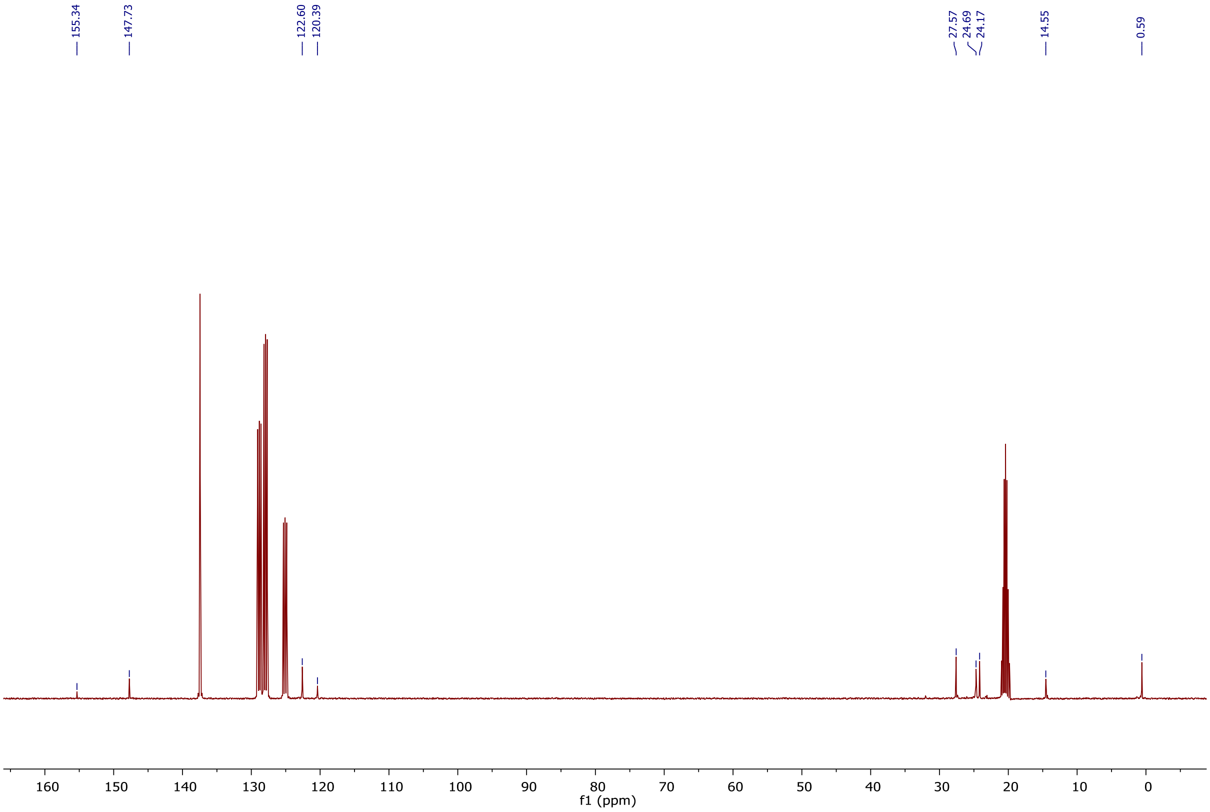

Supplementary Figure 12. ^{13}C{^{1}H} NMR Spectrum (C_6D_8, 298 K, 100.62 MHz) for **$[\{CH_2SiMe_2NDipp\}_2BeClRb]_2$ (9).**

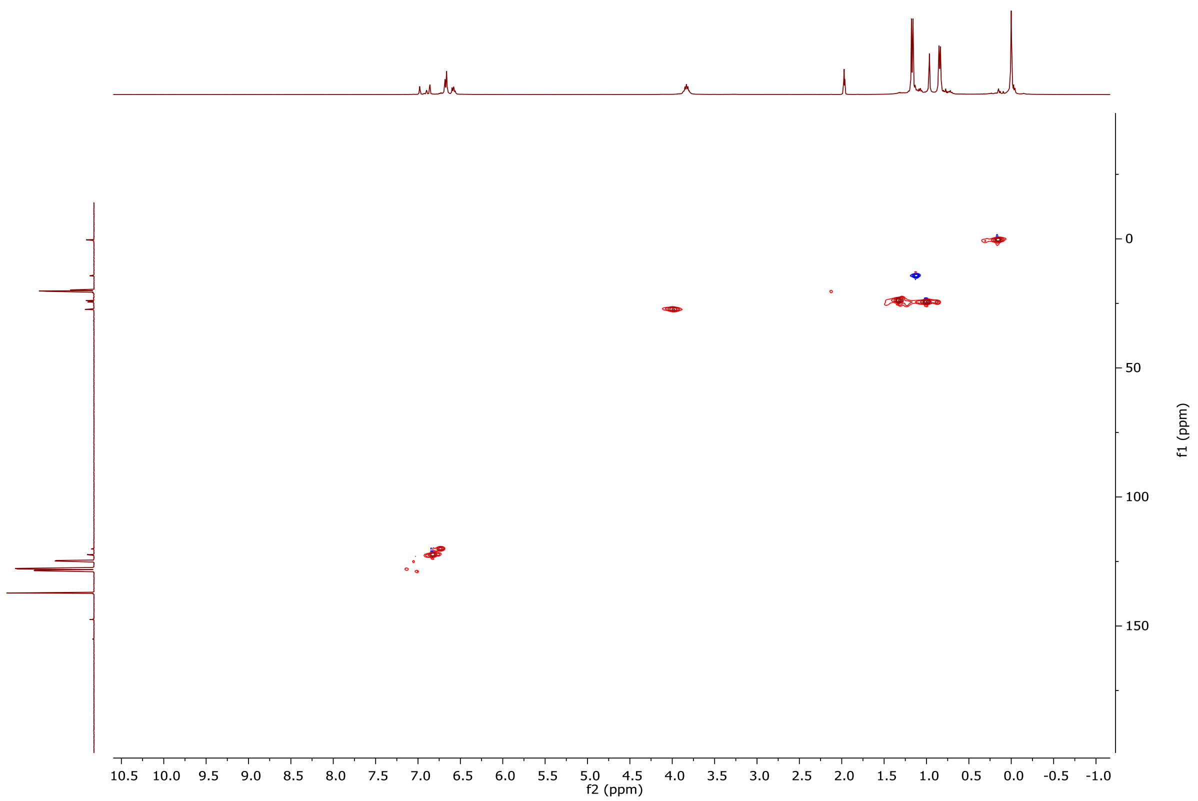

Supplementary Figure 13. ^{1}H-^{13}C HSQC trace (C_6D_8, 298 K, 400.13, 100.62 MHz) for **[{CH$_2$SiMe$_2$NDipp}$_2$BeClRb]$_2$ (9).**

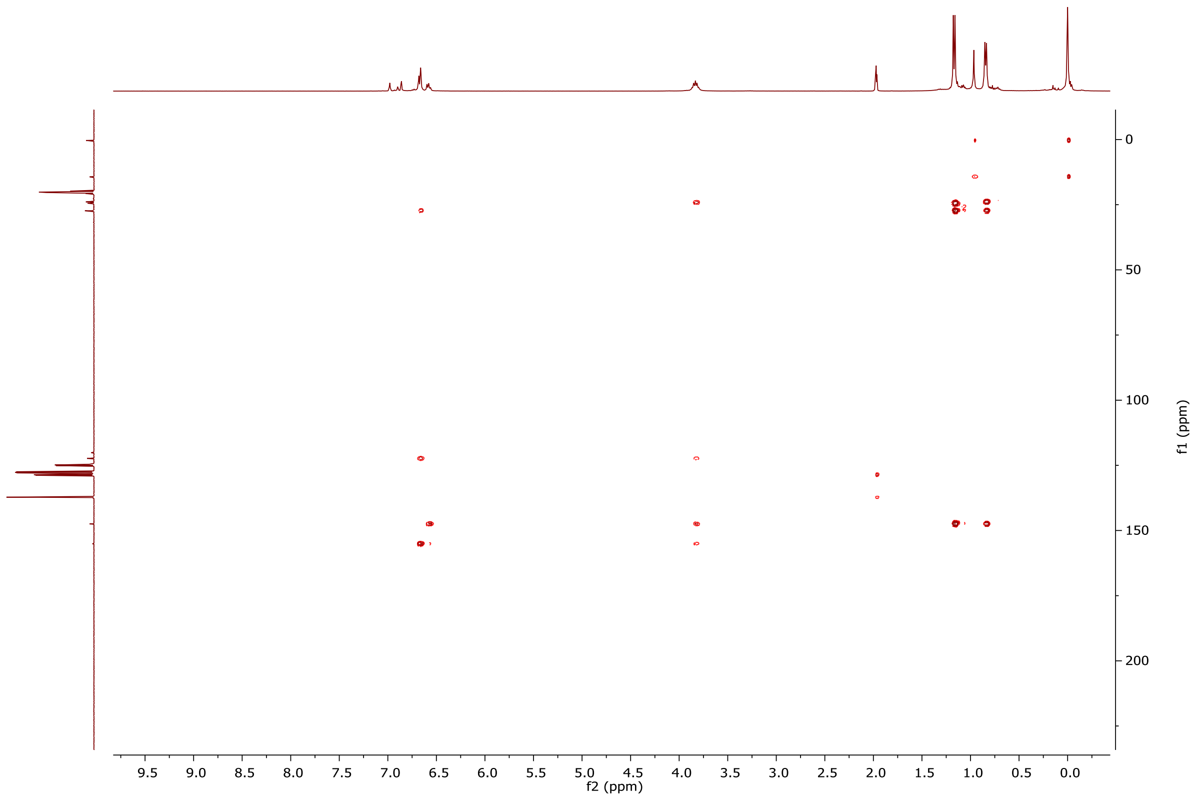

Supplementary Figure 14. ^{1}H-^{13}C HMBC trace (C_6D_8, 298 K, 400.13, 100.62 MHz) for **[{CH$_2$SiMe$_2$NDipp}$_2$BeClRb]$_2$ (9).**

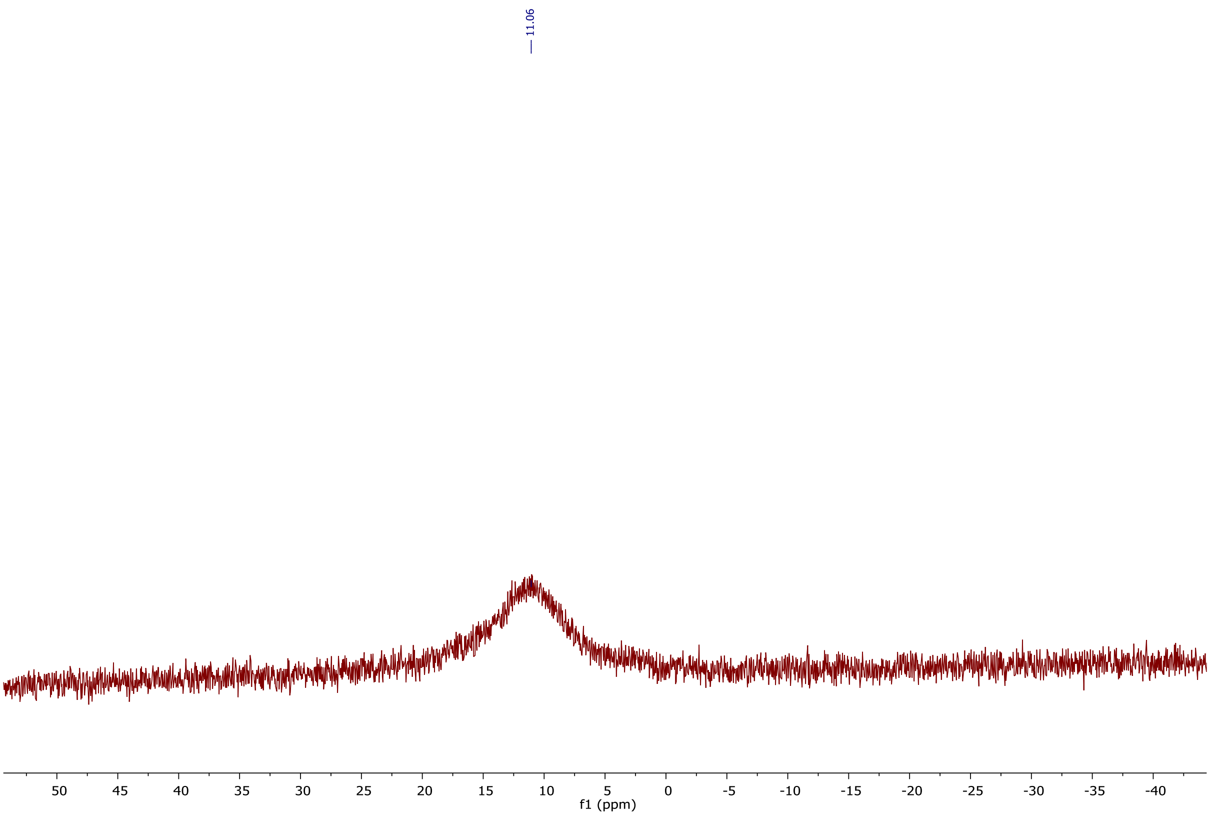

Supplementary Figure 15. ^{9}Be NMR Spectrum (C_6D_8, 298 K, 56.2 MHz) for **[{CH$_2$SiMe$_2$NDipp}$_2$BeClRb]$_2$** (**9**).

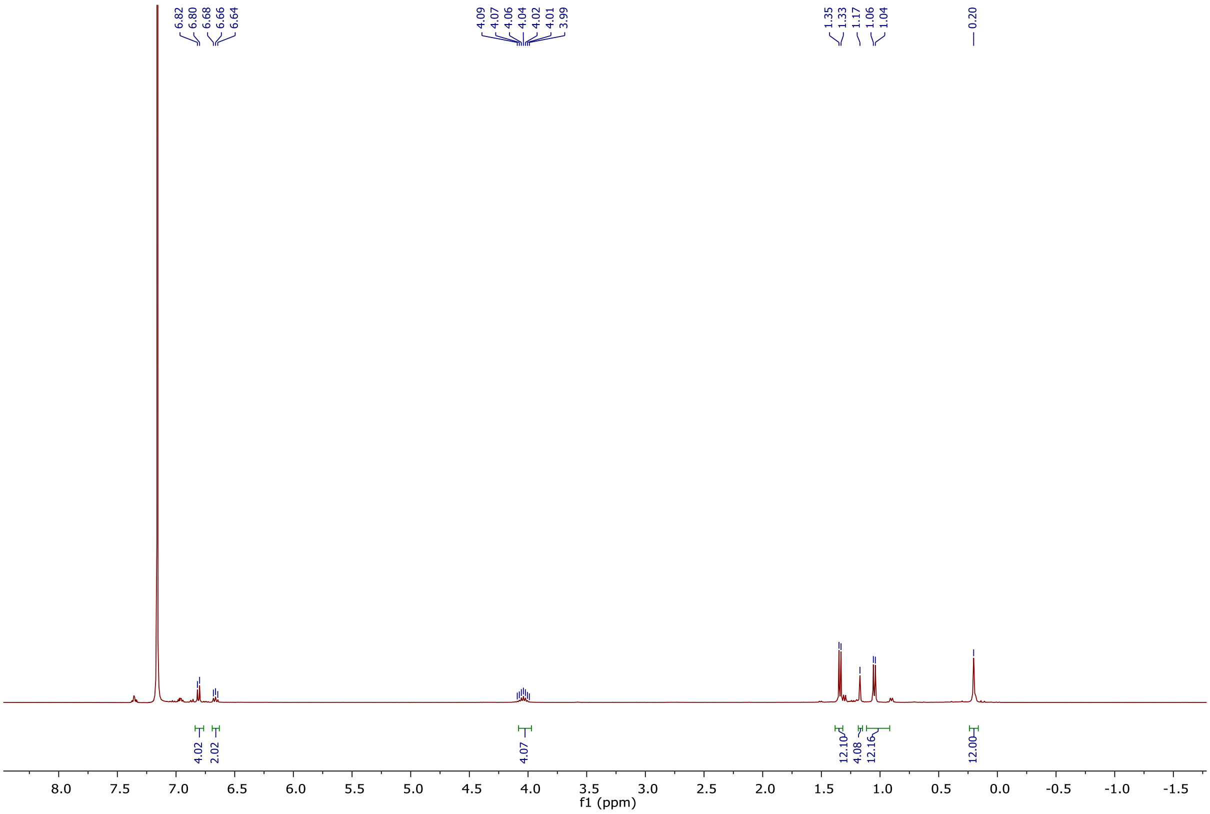

Supplementary Figure 16. ^{1}H NMR Spectrum (C_6D_8, 298 K, 400.13 MHz) for **[{CH$_2$SiMe$_2$NDipp}$_2$BeClCs]$_2$** (**10**).

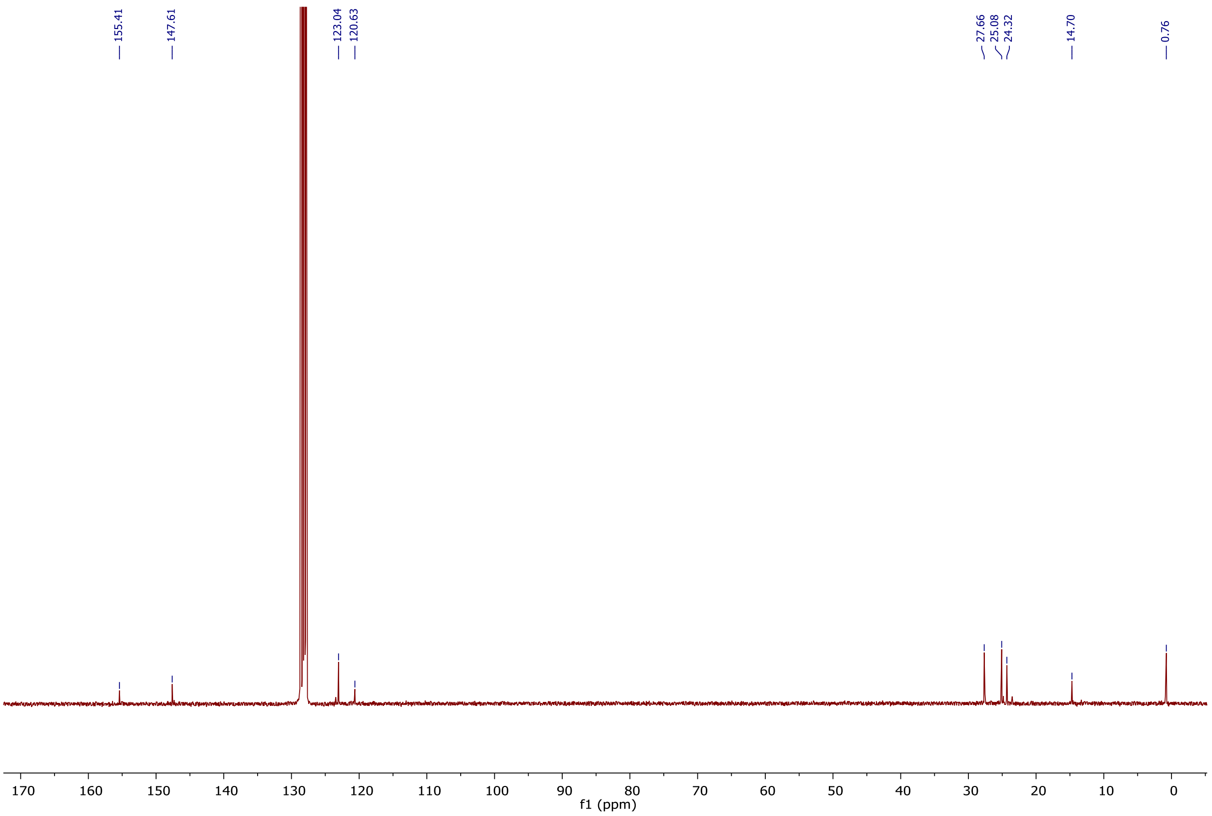

Supplementary Figure 17. $^{13}C\{^{1}H\}$ NMR Spectrum (C_6D_8, 298 K, 100.62 MHz) for **[{CH$_2$SiMe$_2$NDipp}$_2$BeClCs]$_2$** (**10**).

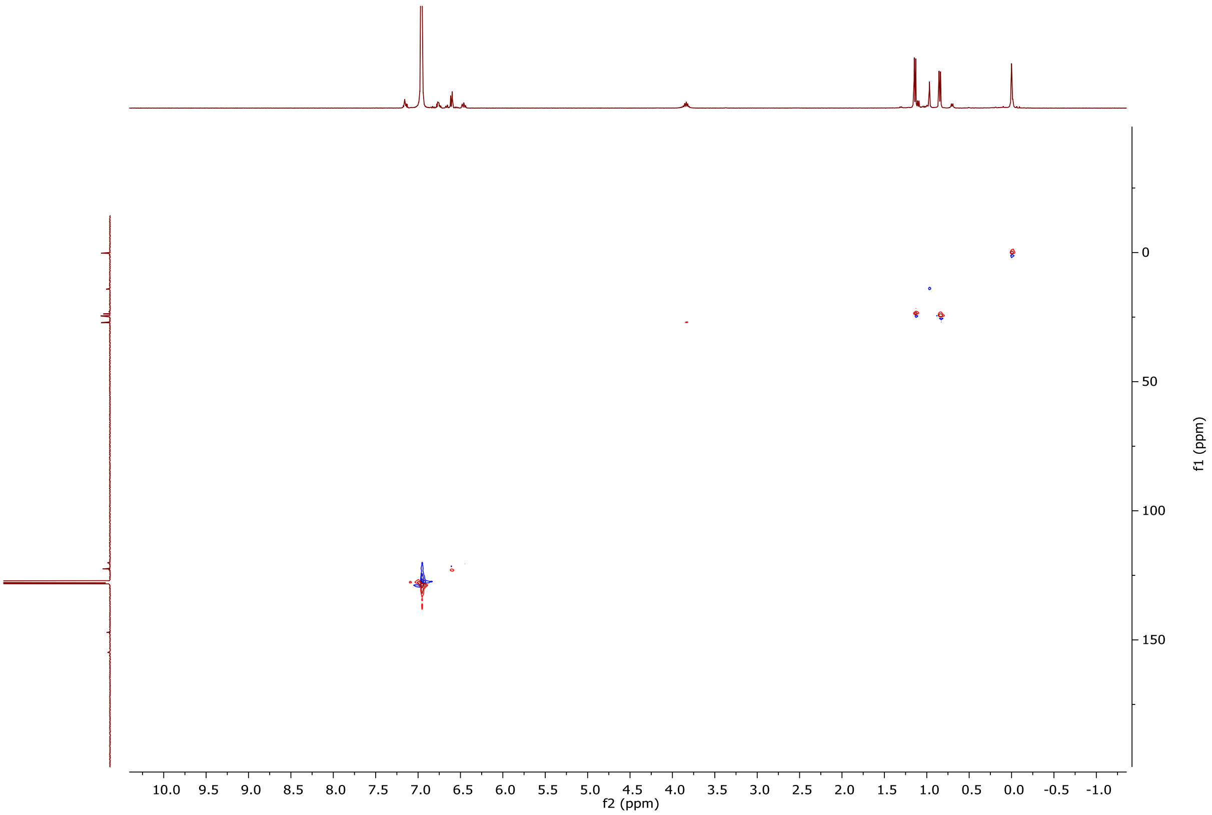

Supplementary Figure 18. ^{1}H-^{13}C HSQC trace (C_6D_8, 298 K, 400.13, 100.62 MHz) for **[{CH$_2$SiMe$_2$NDipp}$_2$BeClCs]$_2$** (**10**).

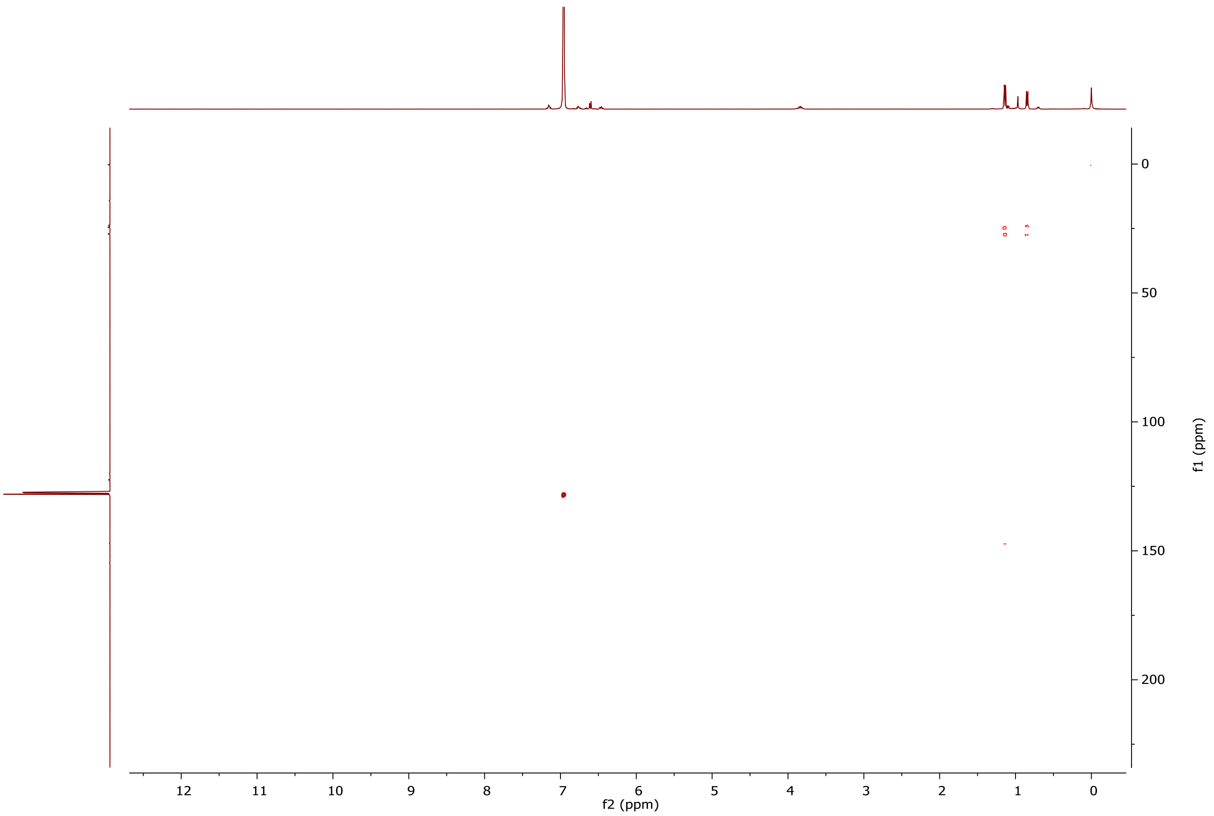

Supplementary Figure 19. ^{1}H-^{13}C HMBC trace (C_6D_8, 298 K, 400.13, 100.62 MHz) for **[{CH$_2$SiMe$_2$NDipp}$_2$BeClCs]$_2$ (10).**

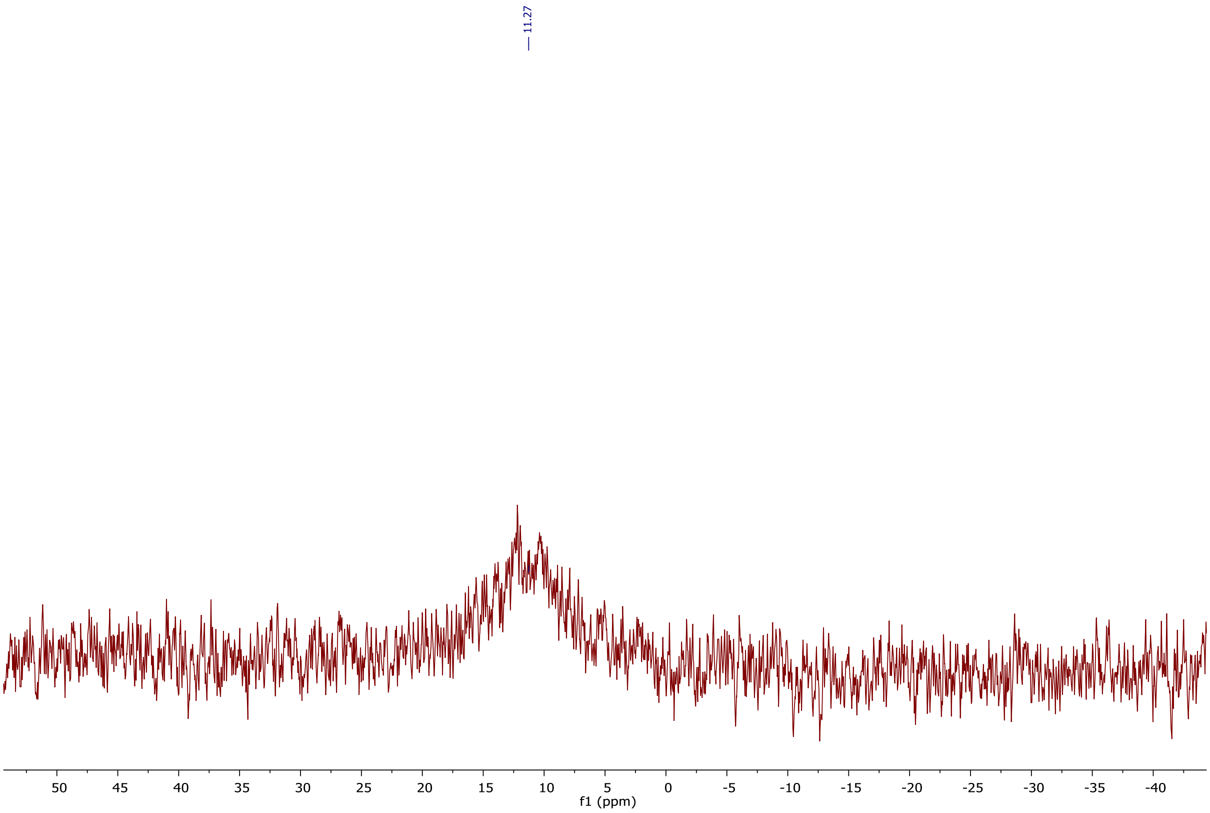

Supplementary Figure 20. ^{9}Be NMR Spectrum (C_6D_6, 298 K, 56.2 MHz) for **[{CH$_2$SiMe$_2$NDipp}$_2$BeClCs]$_2$** (**10**).

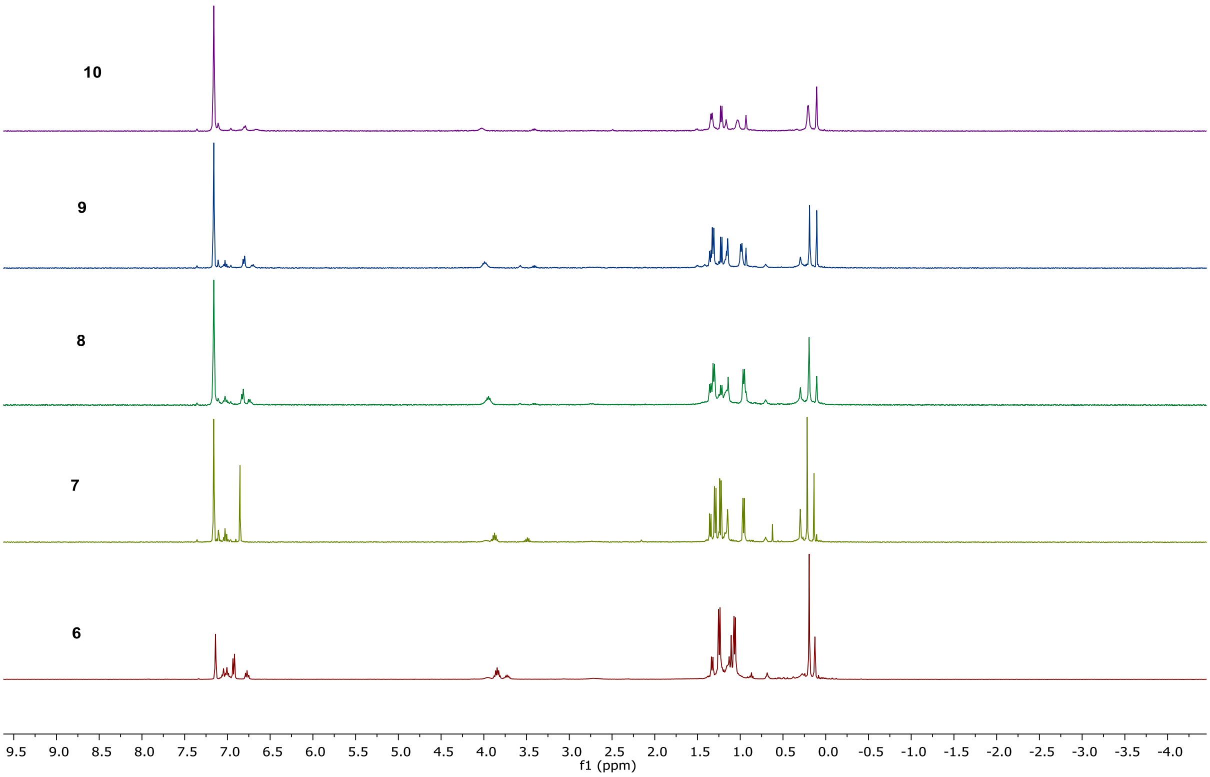

Supplementary Figure 21. ^{1}H NMR Spectrum (C_6D_6, 298 K, 400.13 MHz) for the sequential reduction reactions from **6-10.**

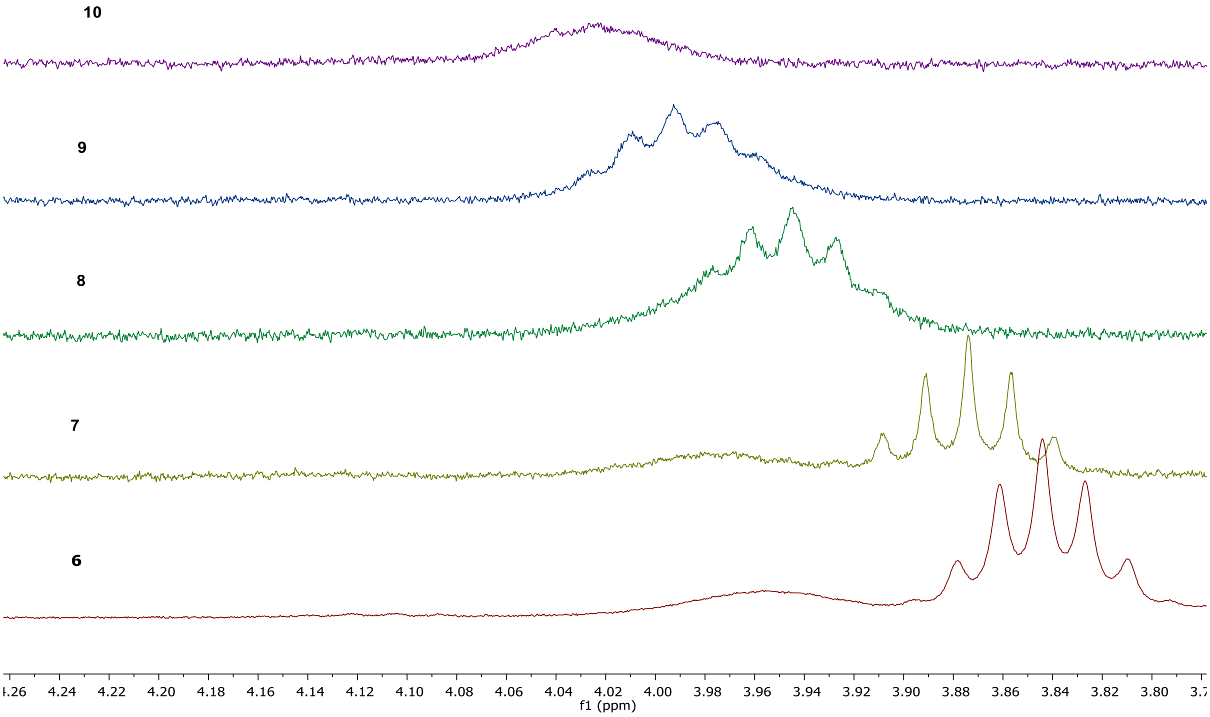

Supplementary Figure 22. ^{1}H NMR Spectrum (C_6D_6, 298 K, 400.13 MHz), Expansion of the Dipp methine resonances in **Figure S21**

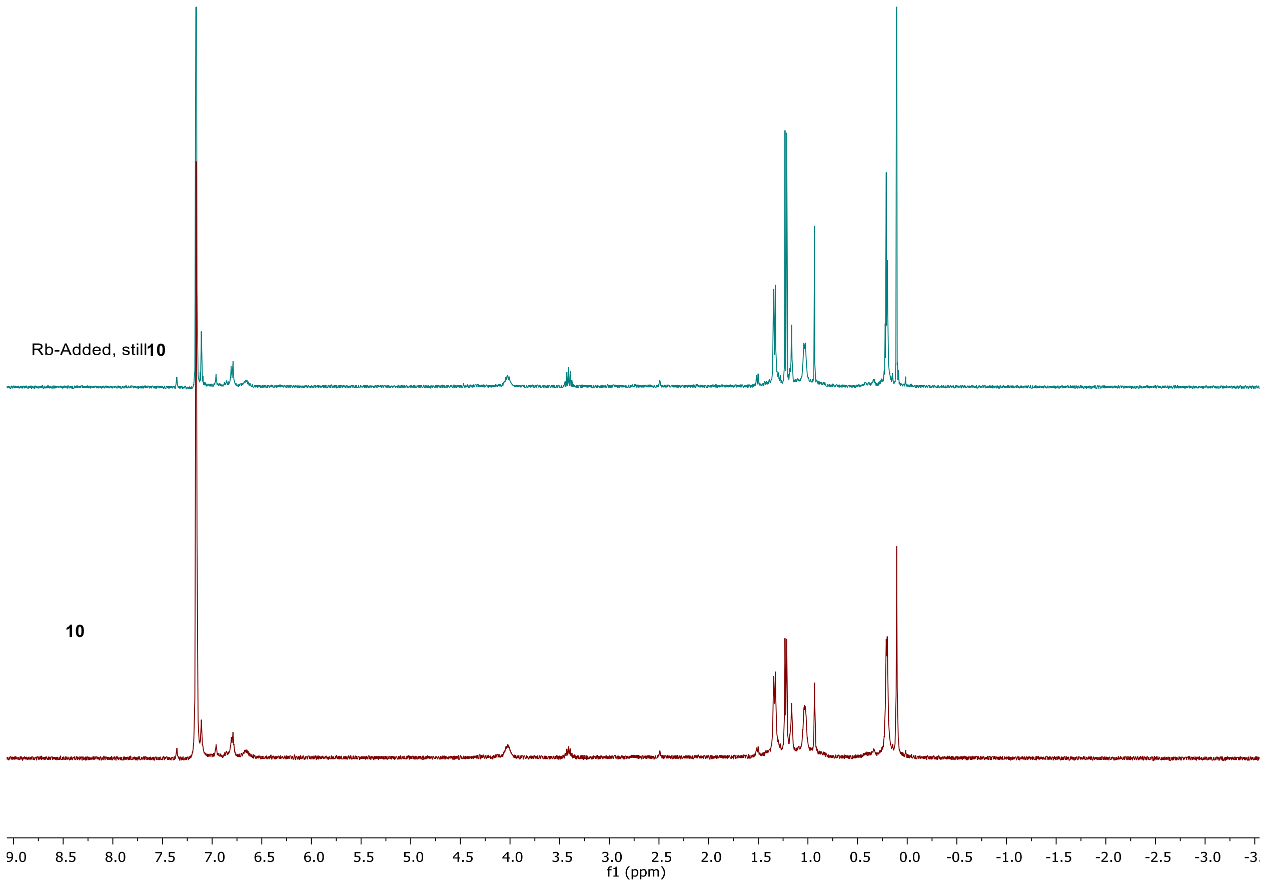

Supplementary Figure 23. ^{1}H NMR Spectrum (C_6D_6, 298 K, 400.13 MHz) for the attempted reduction of **10** to **9**.

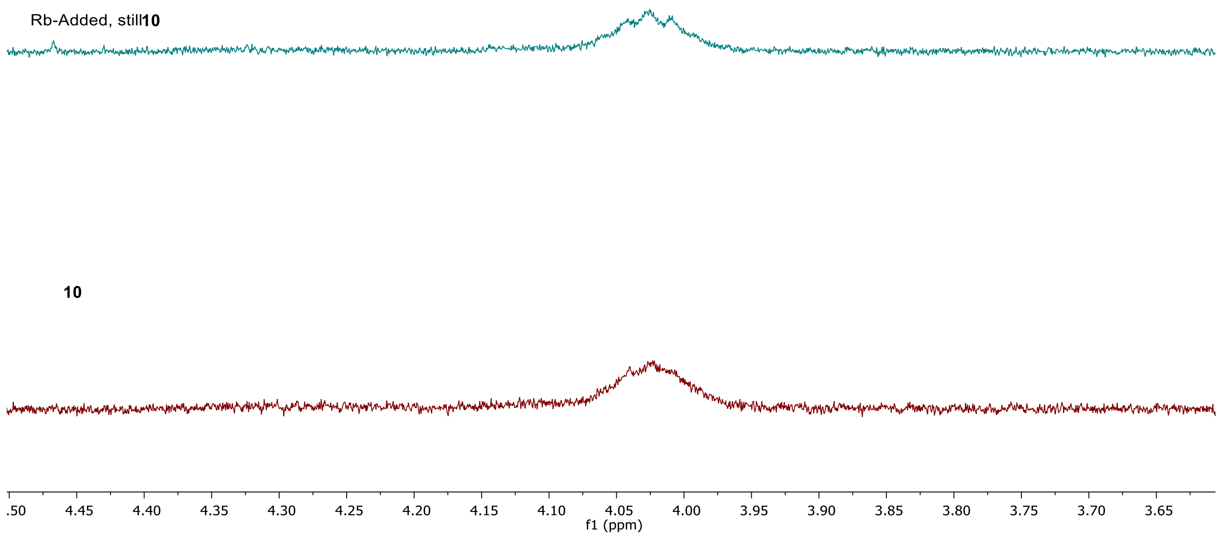

Supplementary Figure 24. ^{1}H NMR Spectrum (C_6D_6, 298 K, 400.13 MHz), Expansion of the Dipp methine resonances in **Supplementary Figure 23**.

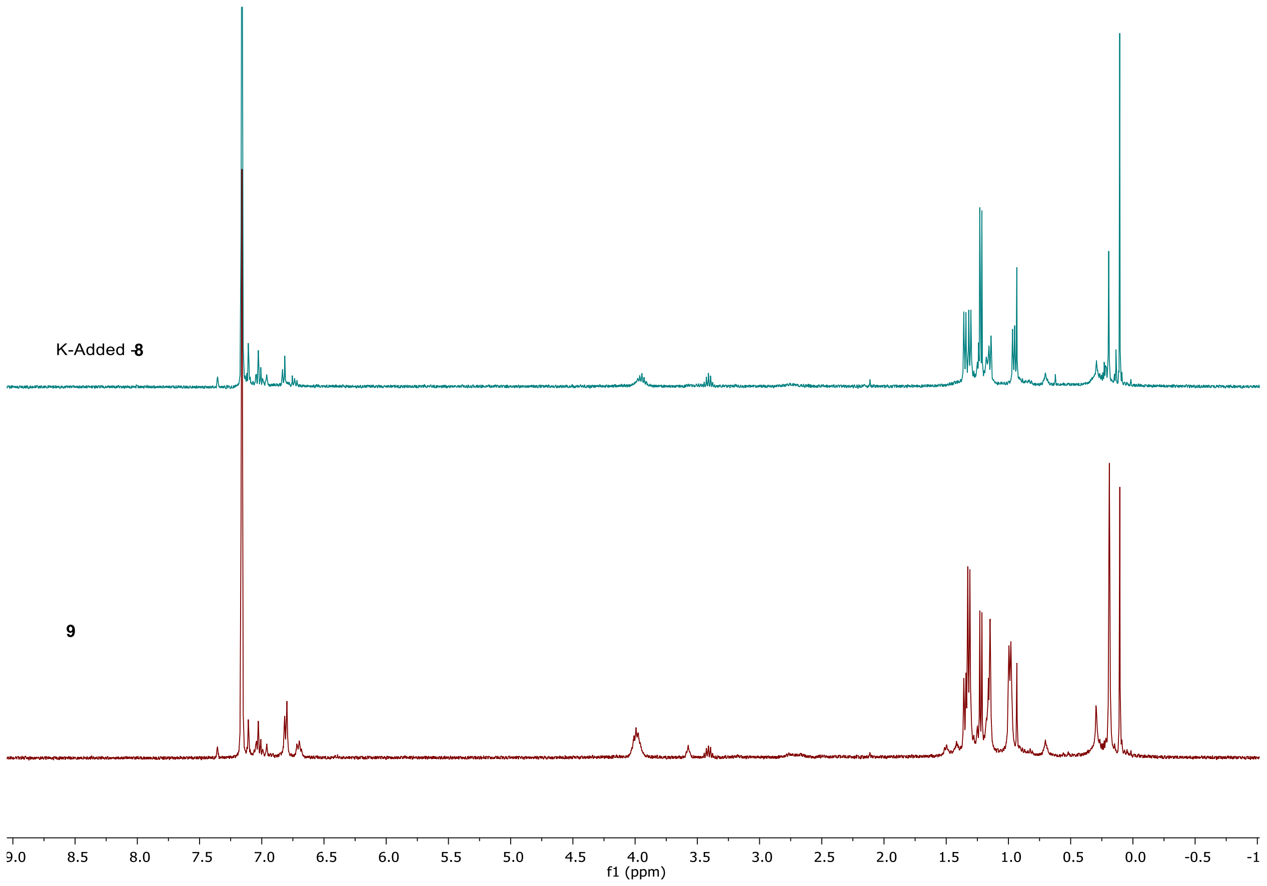

Supplementary Figure 25. ^{1}H NMR Spectrum (C_6D_6, 298 K, 400.13 MHz) for the reduction of **9** to **8**.

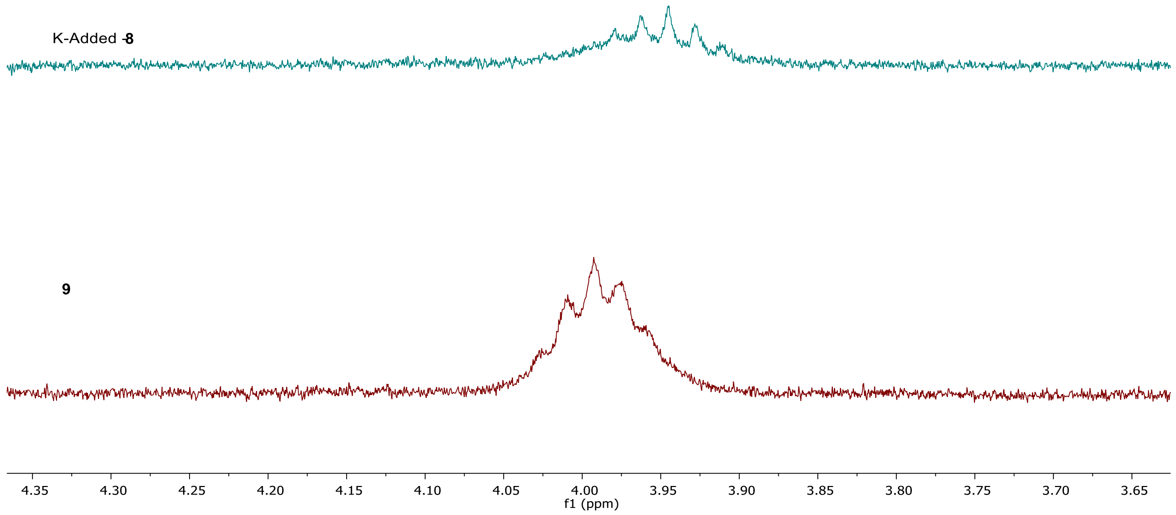

Supplementary Figure 26. ^{1}H NMR Spectrum (C_6D_6, 298 K, 400.13 MHz), Expansion of the Dipp methine resonances in **Figure S25.**

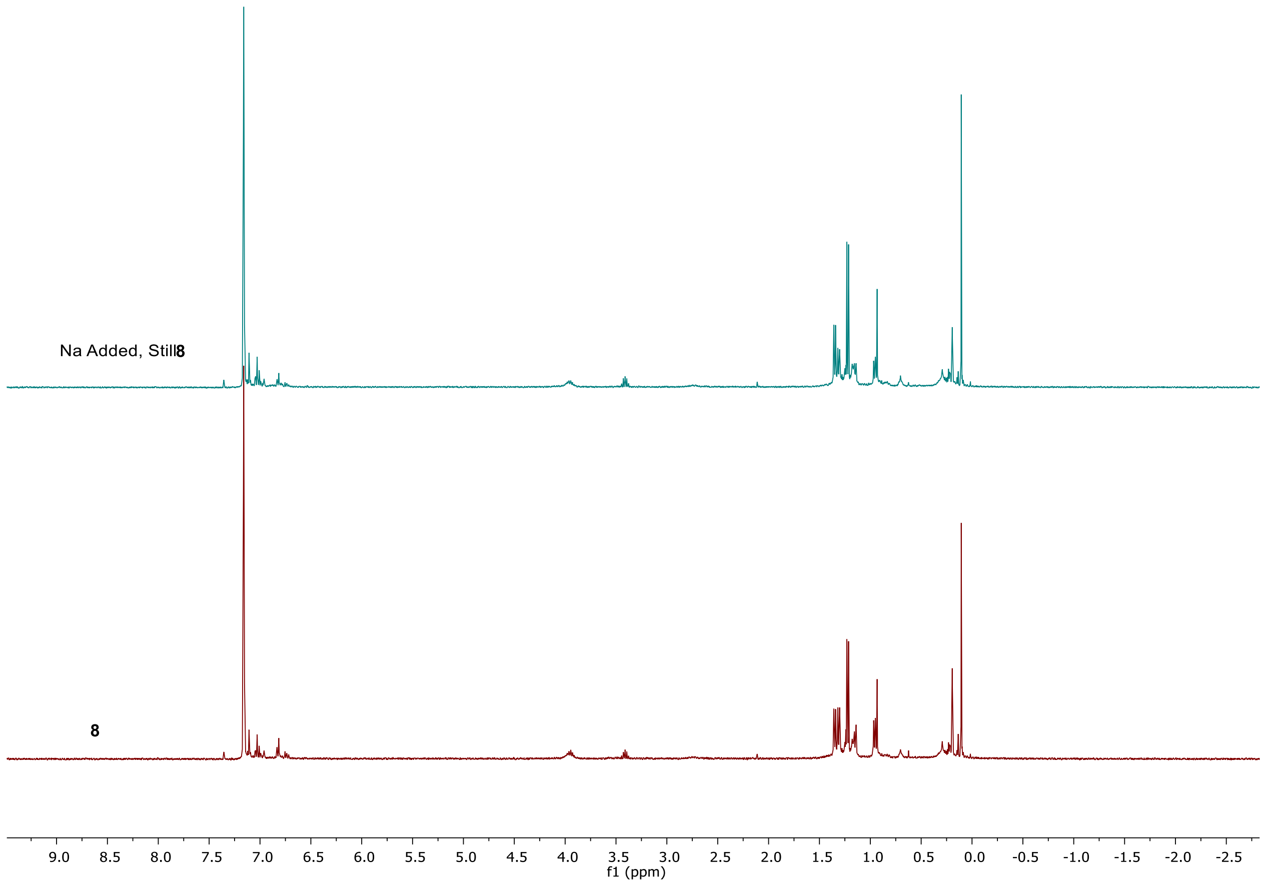

Supplementary Figure 27. ^{1}H NMR Spectrum (C_6D_6, 298 K, 400.13 MHz) for the attempted reduction of **8** to **7**.

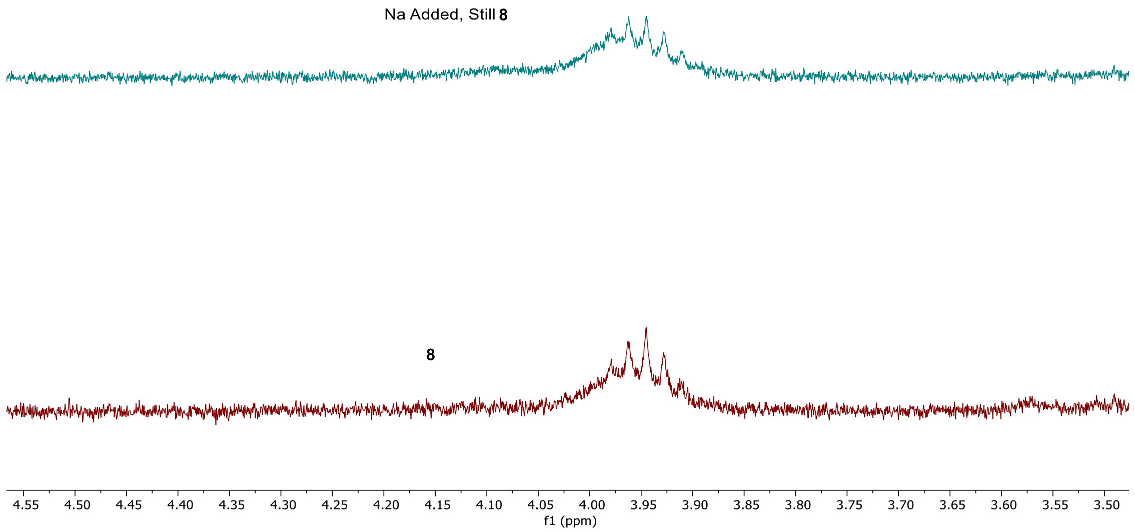

Supplementary Figure 28. ^{1}H NMR Spectrum (C_6D_6, 298 K, 400.13 MHz), Expansion of the Dipp methine resonances in **Supplementary Figure 27.**

Crystallographic Details

Single Crystal X–ray diffraction data for compounds **7 - 10** were collected on a SuperNova EosS2 diffractometer using CuKα (λ = 1.54184 Å) radiation. The crystals were maintained at 150 K during data collection. Using Olex2,[4] the structures were solved with ShelXT and refined with the ShelXL[6] refinement package using Least Squares minimisation. Distance and ADP restraints pertaining to fractional-occupancy atoms were included on merit, throughout, to assist convergence.

The asymmetric unit in **7** contains one molecule of the complex and half of one molecule of hexane. C61 and C62 of the latter were treated for 70:30 disorder.

The hydrogen atoms attached to C26 have been located and refined, at a distance of 0.98 Å from the parent carbon, in the structure of **8**. There is also one molecule of benzene present, per asymmetric unit.

In **9**, the asymmetric unit comprises two crystallographically independent dimers plus one molecule of benzene. Both the rubidium centre and the isopropyl group based on C25 were treated for 60:40 disorder. The ADP pertaining to Cl1 is also suggestive of some disorder, but efforts to model same did not converge well without excessive restraints and, hence, this was abandoned. In the fragment based on Be2, the halide was successfully modelled to take account of 60:40 disorder.

In **10** there is half of a dimer molecule in the asymmetric unit plus one benzene molecule. The remainder of the complex is generated via the inversion symmetry intrinsic to space group $P2_1/n$. C15 and C16 were treated for 65:35 disorder.

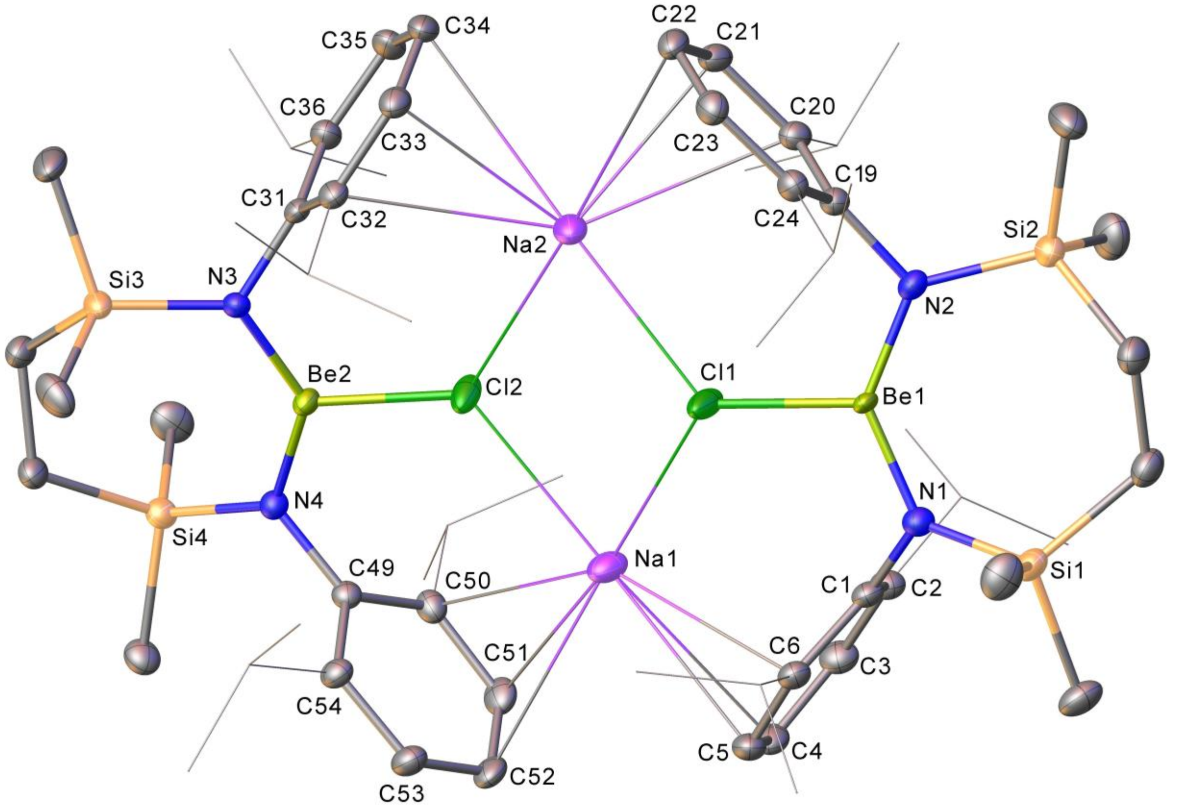

Supplementary Figure 29: Displacement ellipsoid plot (30% probability) of compound **7** (CCDC 2293577).

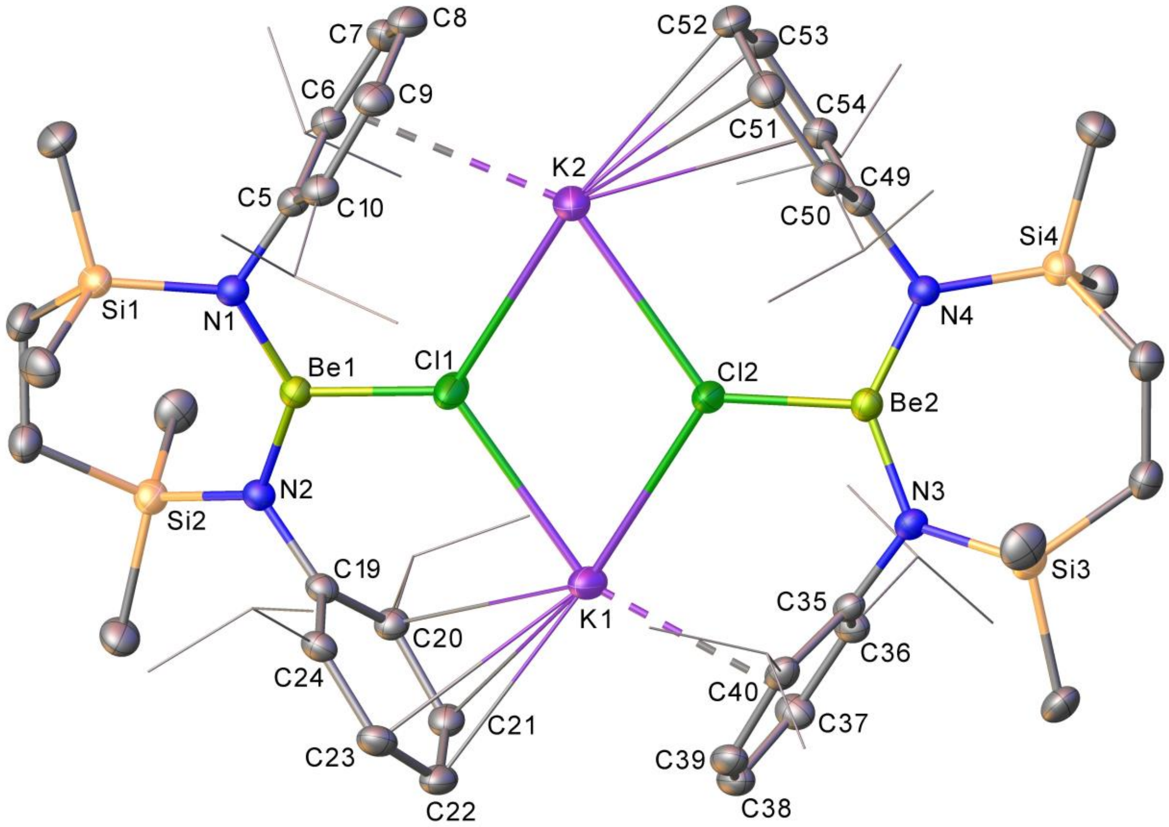

Supplementary Figure 30: Displacement ellipsoid plot (30% probability) of compound **8** (CCDC 2293578).

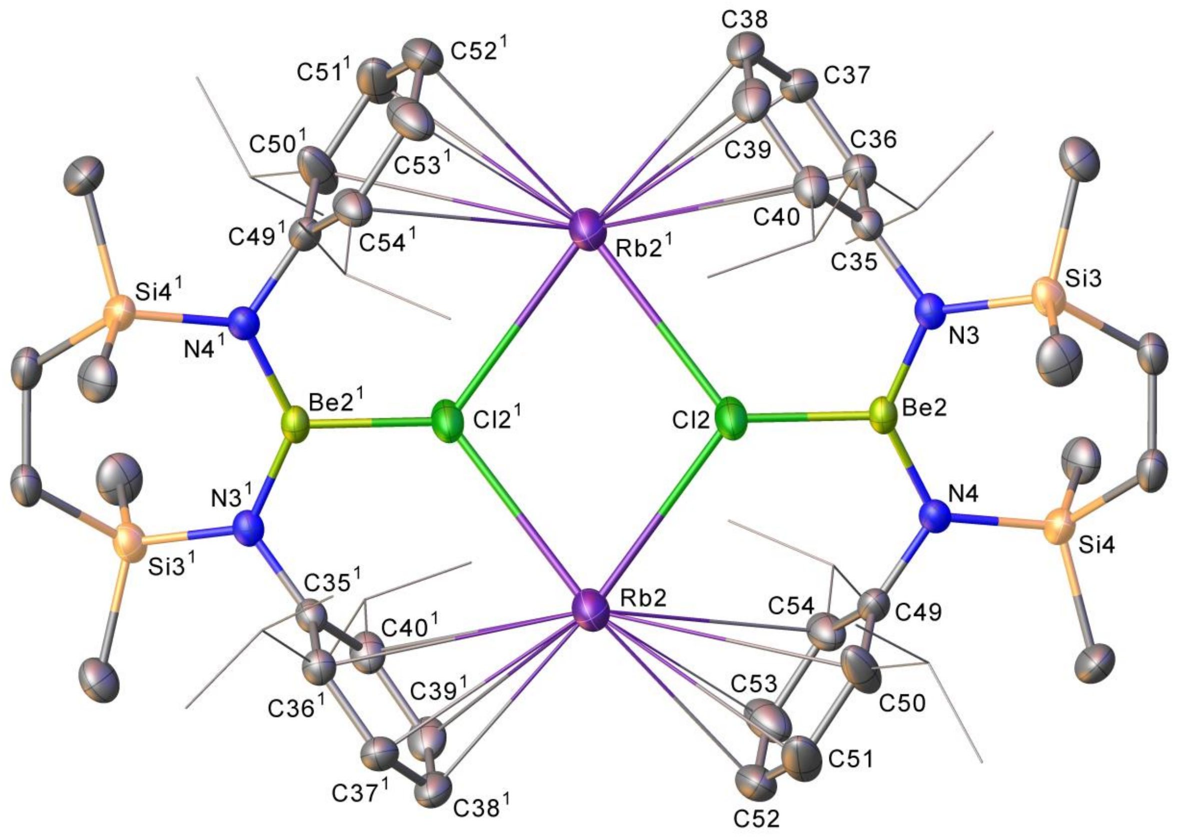

Supplementary Figure 31: Displacement ellipsoid plot (30% probability) of compound **9** (CCDC 2293579).

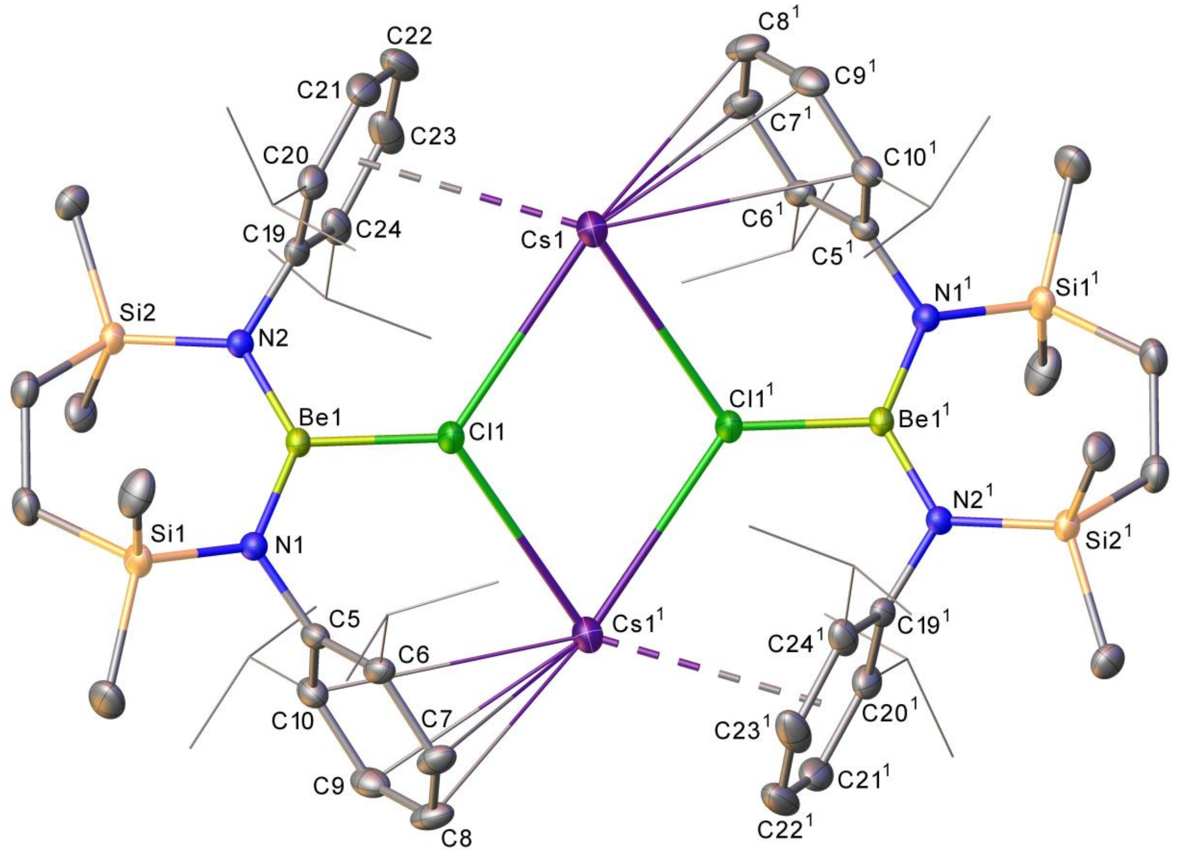

Supplementary Figure 32: Displacement ellipsoid plot (30% probability) of compound **10** (CCDC 2293580).

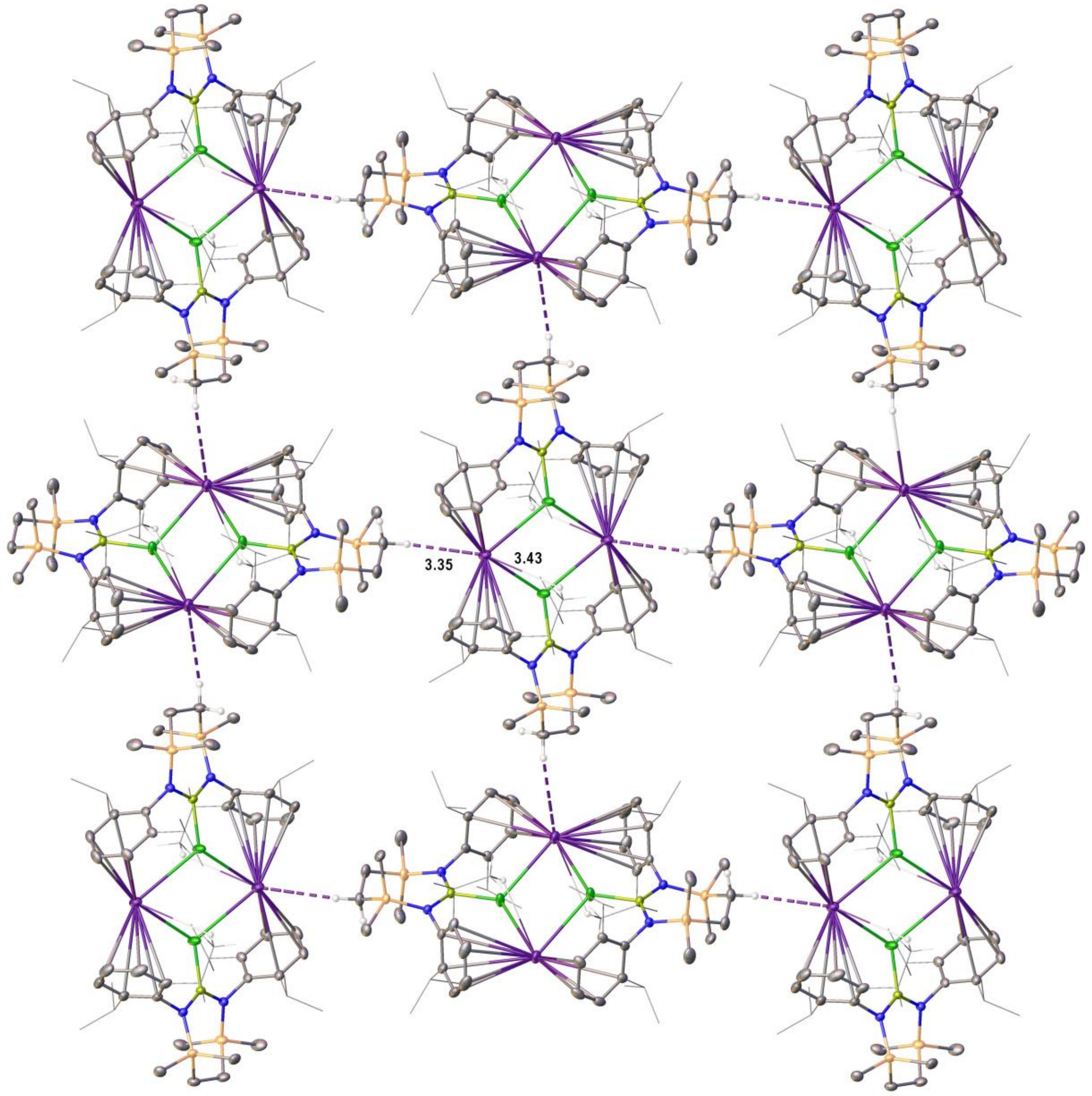

Supplementary Figure 33. Displacement ellipsoid plot (30% probability) of a polymeric section of compound **10**. Hydrogen atoms, apart from H30A and H2A, and a molecule of benzene solvent are omitted and, for clarity, iso-propyl substituents are shown as wireframe.

Supplementary Table 1: Crystal data and structure refinement for compounds **7 - 10**.

Identification code	**7**	**8**	**9**	**10**
Empirical formula	$C_{63}H_{107}Be_2Cl_2N_4Na_2Si_4$	$C_{66}H_{106}Be_2Cl_2K_2N_4Si_4$	$C_{66}H_{106}Be_2Cl_2N_4Rb_2Si_4$	$C_{72}H_{112}Be_2Cl_2Cs_2N_4Si_4$
Formula weight	1167.78	1235.02	1327.76	1500.75
Crystal system	triclinic	monoclinic	monoclinic	monoclinic
Space group	*P*-1	$P2_1/c$	*C*2/*c*	$P2_1/n$
a/ Å	10.2008(4)	32.2961(7)	43.3154(9)	12.11433(10)
b/ Å	12.6724(4)	10.3658(2)	10.6999(2)	19.70079(16)
c/ Å	29.8145(7)	22.4227(5)	32.7332(7)	16.80914(13)
α/ °	94.255(2)	90	90	90
β/ °	95.922(2)	102.166(2)	100.3234(19)	96.0821(7)
γ/ °	111.255(3)	90	90	90
U/ $Å^3$	3546.8(2)	7338.0(3)	14925.3(6)	3989.12(5)
Z	2	4	8	2
ρ_{calc}/ g cm^{-3}	1.093	1.118	1.182	1.249
μ/ mm^{-1}	1.866	2.719	3.253	8.605
F(000)	1266.0	2664.0	5616.0	1560.0
Crystal size/ mm^3	0.107 × 0.075 × 0.062	0.305 × 0.093 × 0.065	0.18 × 0.108 × 0.071	0.211 × 0.1 × 0.1
2θ range for data collection/°	7.54 to 146.326	7.962 to 145.806	8.3 to 141.018	6.936 to 146.262
Index ranges	-12 ≤ h ≤ 12<br>-15 ≤ k ≤ 11<br>-34 ≤ l ≤ 36	-39 ≤ h ≤ 39<br>-10 ≤ k ≤ 12<br>-27 ≤ l ≤ 27	-52 ≤ h ≤ 52<br>-13 ≤ k ≤ 10<br>-39 ≤ l ≤ 39	-14 ≤ h ≤ 14<br>-24 ≤ k ≤ 23<br>-20 ≤ l ≤ 16
Reflections collected	28638	58250	75875	28599
Independent reflections, R_{int}	13915, 0.0344	14484, 0.0513	14177, 0.0456	7943, 0.0302
Data/restraints/parameters	13915/41/738	14484/3/756	14177/107/821	7943/24/450
Goodness–of–fit on F^2	1.021	1.024	1.026	1.046
Final *R*1, *wR*2 [*I*>=2σ(*I*)]	0.0478, 0.1144	0.0588, 0.1504	0.0514, 0.1301	0.0388, 0.1023
Final *R*1, *wR*2 [all data]	0.0634, 0.1248	0.0775, 0.1650	0.0640, 0.1403	0.0400, 0.1033
Largest diff. peak/hole/ e $Å^{-3}$	0.54/−0.76	0.81/−0.49	0.57/−0.96	1.71/−0.86

Computational Details

C1. DFT calculations were performed with Gaussian 16 (A.03).[7] The Na, Si, Cl, K, Rb and Cs centres were described with the Stuttgart relativistic effective core potentials (RECPs) and associated basis sets,[8] and the 6-31G** basis set was used for all other atoms (BS1).[9] A polarization function was also added to Si (ζ_d = 0.284), Cl (ζ_d = 0.640), K (ζ_d = 1.000), Rb (ζ_d = 0.491) and Cs (ζ_d = 0.306).[10] Initial BP86 optimizations were performed using the 'grid = ultrafine' option,[11] with all stationary points being fully characterized via analytical frequency calculations as minima with all positive eigenvalues. (Note, that for the structures of (C_6H_6)**Li^+**···C_6H_6, (C_6H_6)**Na^+**···C_6H_6, (C_6H_6)**Rb^+**···C_6H_6 and (C_6H_6)**Cs^+**···C_6H_6, the presence of small imaginary modes (> –10 cm^{-1}) persisted despite attempts to remove them, including re-optimization with 'very tight' convergence criteria and moving the geometry with respect to the imaginary modes). All energies were recomputed with a larger basis set featuring 6-311++G** basis sets on all atoms (BS2), except for Rb and Cs, where the Stuttgart RECPs were again employed with the aforementioned additional polarization functions. Corrections for the effect of benzene (ε = 2.2706) solvent were introduced using the polarizable continuum model and BS1.[12] Single-point dispersion corrections to the BP86 results employed Grimme's D3 parameter set with Becke-Johnson damping as implemented in Gaussian (D3BJ).[13] Further single point calculations were performed for the basis set testing. These include; def2-SVPX for all atoms (including the corresponding ECPs)[14] def2-TZVPPX, Sapporo-DZ-2012X,[15] Sapporo-TZ-2012 (however, both Sapporo basis sets are not available for Cs). Additional single point calculations were performed with ORCA 5.0.1,[16] using the ZORA-def2-TZVPP for all atoms, except Rb and Cs, for which the all-electron ZORA(-SARC-)TZVPP basis set was used,[17] including CPCM solvation, D3BJ dispersion, and the ZORA relativistic approach.[18]

C2. Basis Set Testing of M^+···C_6H_6 Interactions.

Basis set testing was carried out to discern whether an effective core potential or an all-electron basis set was more appropriate to describe the alkali metal centres in the $[Be]_2M_2$ structures, **7** – **10**, resulting from reductive de-lithiation of **6**. Given that all resulting complexes are considered to feature stabilizing cation-arene interactions with the group 1 metal centres, we considered it appropriate to benchmark against a series of $[M(C_6H_6)_n]^+$ systems (n = 1 – 2), the experimental bond dissociation energies (BDEs, ΔE) of which have been measured by gas-phase collision-induced dissociation (CID) experiments, and reported by Armentrout and co-workers.[19] In their study, Armentrout identified that the use of RECPs to describe the heavier group 1 elements led to divergent BDEs with respect to experimental data. The results of the basis set testing of these systems, at both the BP86-D3BJ/BS2//BP86/BS1 and BP86-D3BJ/ZORA(-SARC)-def2-TZVPP//BP86/BS1 levels of theory (simply expressed as 'ZORA' in Supplementary Table 2), are presented below in Supplementary Table 2.

Supplementary Table 2. Experimental and computational bond dissociation energies, in kJ/mol.

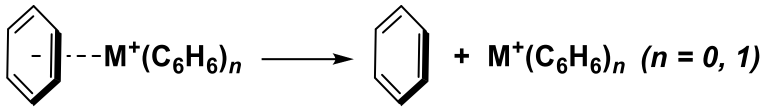

Entry	Species	Experiment	BS2	*Error (BS2)*	ZORA	*Error (ZORA)*
**1**	**Li**$^+\cdots C_6H_6$	–161.1	–156.4	*4.7*	–154.2	*6.9*
**2**	**Na**$^+\cdots C_6H_6$	–92.6	–103.7	*–11.1*	–102.5	*–9.9*
**3**	**K**$^+\cdots C_6H_6$	–73.3	–77.2	*–3.9*	–74.7	*–1.4*
**4**	**Rb**$^+\cdots C_6H_6$	–68.5	–64.9	*3.6*	–69.5	*–1.0*
**5**	**Cs**$^+\cdots C_6H_6$	–64.6	–60.8	*3.8*	–61.9	*2.7*
**6**	(C_6H_6)**Li**$^+\cdots C_6H_6$	–104.2	–109.9	*–5.7*	–109.0	*–4.8*
**7**	(C_6H_6)**Na**$^+\cdots C_6H_6$	–80.0	–89.1	*–9.1*	–86.5	*–6.5*
**8**	(C_6H_6)**K**$^+\cdots C_6H_6$	–67.5	–66.5	*1.0*	–64.8	*2.7*
**9**	(C_6H_6)**Rb**$^+\cdots C_6H_6$	–62.7	–58.5	*4.2*	–59.3	*3.4*
**10**	(C_6H_6)**Cs**$^+\cdots C_6H_6$	–58.8	–53.4	*5.4*	–52.3	*6.5*
	Mean Absolute Deviation	*N/A*	*5.4*		*4.8*	

While the performance of the BS2 and 'ZORA' basis sets, i.e. an ECP and all-electron approach respectively, are incredibly similar when it comes to the cation-arene interactions of the lighter group 1 elements, a more pronounced difference is seen with the heavier elements (K – Cs). ZORA is in better agreement to experiment than BS2 (with the exception of $(C_6H_6)Cs^+\cdots C_6H_6$, entry 10). While only a small difference in overall performance was observed for the two different basis set approaches, the all-electron approach with ZORA(-SARC)-def2TZVPP is a slightly better performer when accounting for cation···arene interactions with a mean absolute deviation (MAD) of 4.8 kJ mol^{-1}.

C3. Chemical Models Employed in Reduction Calculations.

C3.1. Atomic Alkali Metal Reductant.

The first chemical model used to calculate the free energies of reduction of **6** with Na, K, Rb and Cs assumed the group 1 metal reductants were solvent separated atoms in solution in a doublet spin-state. Supplementary Table 3 presents the computed free energies using this approach.

Supplementary Table 3. Computed lithium reduction energies (ΔG_{bnz} / kcal mol $^{-1}$) with alkali metals, using atomic M(0) (see Section 1 of computational details).

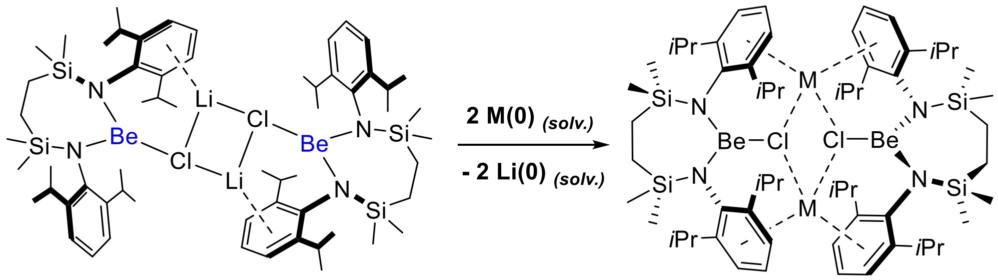

6 **7** (M = Na), **8** (K), **9** (Rb), **10** (Cs)

**2 M(solv)**	***ΔGbnz / kcal mol −1***					
	ECP on Rb, Cs			***all electron***		
		**Def2**		**Sapporo**		
**M**	**BS2***	**SVP**	**TZVPP**	**DZ**	**TZ**	**ZORA-(SARC)-def2-TZVPP**
**Na (7)**	−0.2	−4.0	+1.4	−4.6	−0.5	+6.1
**K (8)**	−9.4	−9.7	−9.5	−11.3	−10.2	−0.7
**Rb (9)**	+0.2	+0.8	−1.1	−8.2	−8.0	+3.3
**Cs (10)**	−3.1	0.0	−4.3	N/A	N/A	+1.9

****BS2*** = *SDDAll = Rb & Cs, 6-311++G** = H, C, N, Si, Be, Cl, Li, Na, K.*

C3.2. M_9 Cluster as Alkali Metal Reductant.

In the second chemical model constructed to calculate the free energies of reduction of **6**, solution-phase nonameric alkali metal clusters (i.e. M_9) were used in an effort to account for experimental addition of the group 1 metal reductants in their solid state, and with lithium metal extruded in the solid state at the end of each de-lithiation reaction. Each nonameric metal structure was optimized as a doublet (i.e. **$^2[M(0)_9]$**). The resulting computed free energies with this approach are presented in Table S4 and the optimized cluster structures with selected interatomic distances and angles in Supplementary Figure 34.

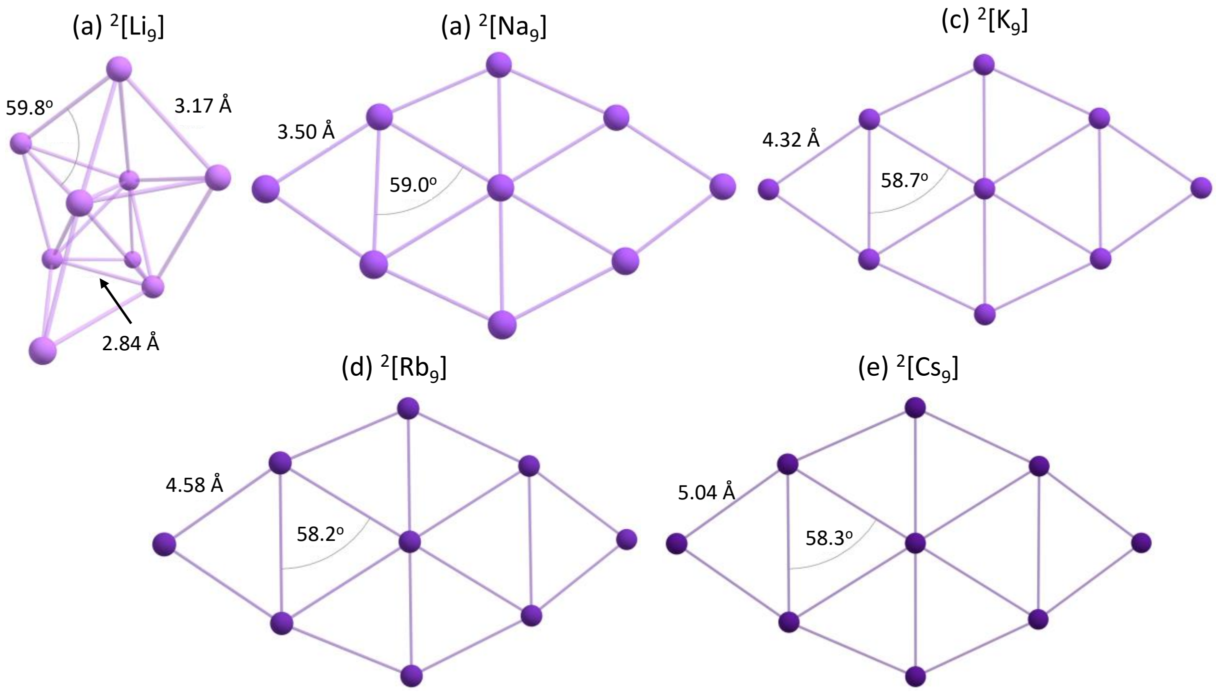

Supplementary Figure 34. Optimized structures of the nonameric alkali metal clusters, $^2[M_9]$, with selected interatomic distances (Å) and bond angles (º).

Supplementary Table 4. Computed lithium reduction energies (ΔG_{bnz} / *kcal mol* $^{-1}$) *with alkali metals, using* M_9 *clusters.*

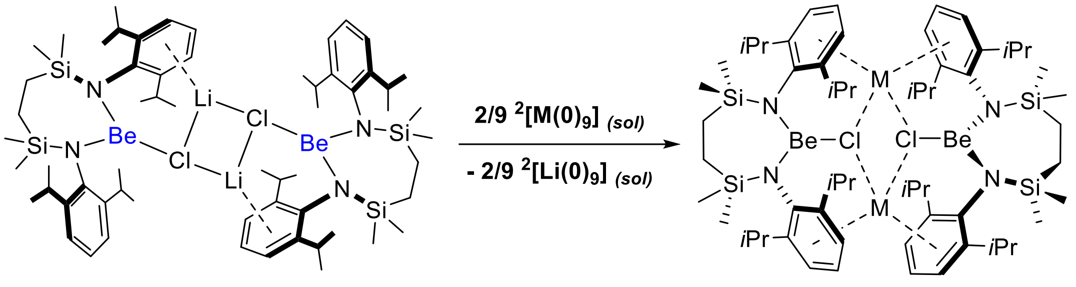

6 — **7** (M = Na), **8** (K), **9** (Rb), **10** (Cs)

$\left(\frac{2}{9}\right)$ $M_{9\ (solv)}$	ΔG_{bnz} / *kcal mol* $^{-1}$					
	ECP on Rb, Cs			***all electron***		
		**Def2**		**Sapporo**		
**M**	**BS2***	**SVP**	**TZVPP**	**DZ**	**TZ**	**ZORA-(SARC)-def2-TZVPP**
**Na (7)**	−11.5	−14.4	−10.8	−13.8	−10.7	−7.2
**K (8)**	−24.1	−23.8	−23.1	−24.2	−23.4	−20.5
**Rb (9)**	−15.8	−15.9	−16.4	−22.5	−22.4	−17.7
**Cs (10)**	−20.2	−18.3	−21.4	N/A	N/A	−20.2

****BS2** = SDDAll = Rb & Cs, 6-311++G** = H, C, N, Si, Be, Cl, Li, Na, K.*

C3.3. Solid-State Alkali Metal Reductant Using a Combined Experimental and Computational Hess Cycle

The third chemical model, uses the first method with solvent separated M(0) atoms (see section C3.1), alongside the incorporation of experimental enthalpies of atomization, with entropies, taken from CODATA[20] to account for the alkali metal in the solid state. Supplementary Figure 35 illustrates a combined computational and experimental free energy cycle. The resulting calculated enthalpies and free energies of the reductive de-lithiation reactions of **6** to form **7** – **10**, with this method, are presented in Supplementary Table 5. A solvation correction was obtained implicitly using the CPCM approach implemented in ORCA 5 for each metal, by computing the electronic energy at the BP86/ZORA(-SARC)-def2TZVPP level and subtracting it from the energy at the BP86,CPCM(Benzene)/ZORA(-SARC)-def2TZVPP level.

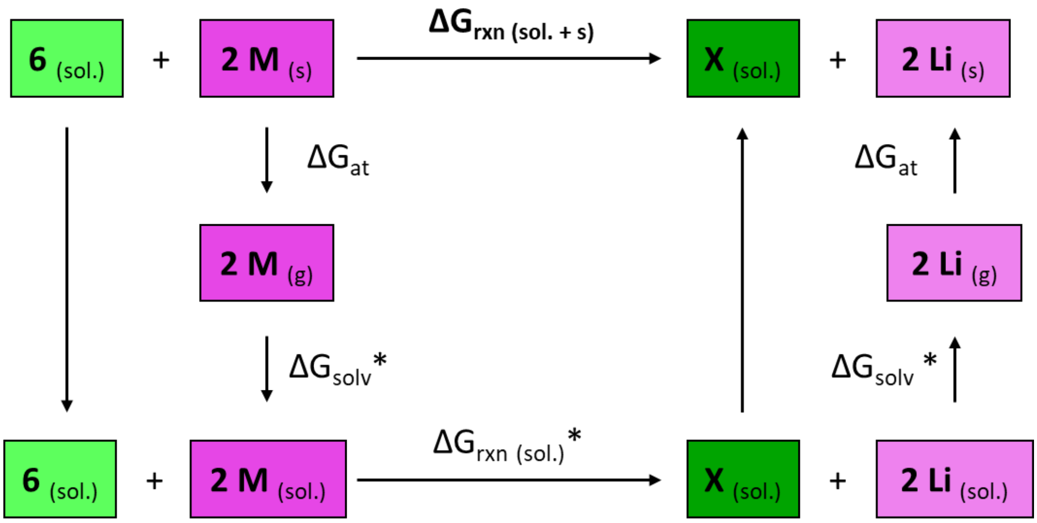

$$\Delta G_{rxn\,(sol.\,+\,s)} = (2 \times \Delta G_{at}[M]) + (2 \times \Delta G_{solv}[M]) + \Delta G_{rxn\,(sol.)} - (2 \times \Delta G_{solv}[Li]) - (2 \times \Delta G_{at}[Li])$$

Supplementary Figure 35. Combined computational and experimental free energy cycle constructed to calculate the reaction free energies of **7** - **10** (* = computed values).

Supplementary Table 5. Thermodynamic values used in the constructed free energy cycle in Supplementary Figure 35, in kJ mol^{-1}. Values taken from reference 20.

M	ΔH_{at}	$\Delta S_{(g)}$	$\Delta S_{(s)}$
**Li**	159.3 ± 1.0	138.782 ± 0.010	29.12 ± 0.20
**Na**	107.5 ± 0.7	153.718 ± 0.003	51.30 ± 0.20
**K**	89.0 ± 0.8	160.341 ± 0.003	64.68 ± 0.20
**Rb**	80.9 ± 0.8	170.094 ± 0.003	76.78 ± 0.30
**Cs**	76.5 ± 1.0	175.601 ± 0.003	85.23 ± 0.40

Supplementary Table 6. Computed lithium reduction energies (ΔG, ΔH / *kcal mol* $^{-1}$) with alkali metals, using the combined computational and experimental Hess cycle as outlined in Section C3.3.

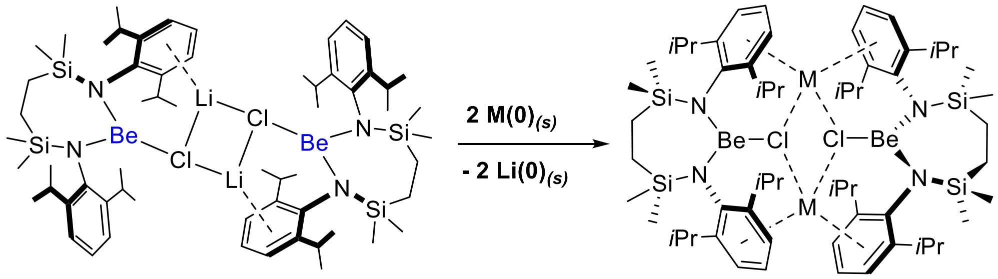

6

7 (M = Na), **8** (K), **9** (Rb), **10** (Cs)

	ZORA(-SARC)-def2-TZVPP	
**M**	**ΔH**	**ΔG**
**Na (7)**	-17.5	-17.6
**K (8)**	-31.7	-32.4
**Rb (9)**	-32.2	-31.9
**Cs (10)**	-34.5	-34.4

C4. Breakdown of Energy Contributions

The Supplementary Tables 7 and 8 detail the evolution of the relative energies as the successive corrections to the initial SCF energy are included. Terms used are:

$\Delta \mathbf{E}_{\mathbf{BS1}}$	SCF energy computed with the BP86 functional with BS1
$\Delta \mathbf{H}_{\mathbf{BS1}}$	Enthalpy at 298.15 K with BS1
$\Delta \mathbf{G}_{\mathbf{BS1}}$	Free energy at 298.15 K and 1 atm with BS1
$\Delta \mathbf{G}_{\mathbf{BS1}/bnz}$	Free energy corrected for benzene solvent with BS1
$\Delta \mathbf{G}_{\mathbf{BS1}/bnz+\mathbf{D3BJ}}$	Free energy corrected for benzene and dispersion effects with BS1
$\Delta \mathbf{E}_{\mathbf{BS2}}$	SCF energy computed with the BP86 functional with BS2
$\Delta \mathbf{G}_{\mathbf{BS2/bnz+D3BJ}}$	Free energy corrected for basis set (BS2), dispersion effects and benzene solvent
$\Delta \mathbf{E}_{\mathbf{ZORA}}$	SCF energy computed with the BP86 functional with ZORA-(SARC)-def2-TZVPP
$\Delta \mathbf{G}_{\mathbf{ZORA/bnz+D3BJ}}$	Free energy corrected for basis set (ZORA-(SARC)-def2-TZVPP), dispersion effects and benzene solvent

Supplementary Table 7. Breakdown of contributions to the overall free energies of de-lithiation of **6** to form **7** – **10** (kcal mol^{-1}) using atomic M(0).

	ΔE_{BS1}	ΔH_{BS1}	ΔG_{BS1}	$\Delta G_{BS1/bnz}$	$\Delta G_{BS1/bnz+D3BJ}$	ΔE_{BS2}	$\Delta G_{BS2/bnz+D3BJ}$	$\Delta E_{ZORA/bnz+D3BJ)}$	$\Delta G_{ZORA/bnz+D3BJ}$
**Na (7)**	22.0	20.9	19.7	12.8	-2.1	24.0	-0.2	8.4	6.1
**K (8)**	19.8	18.4	15.8	8.6	0.0	10.5	-9.4	3.3	-0.7
**Rb (9)**	26.8	25.4	23.4	15.2	6.7	20.3	0.2	6.7	3.3
**Cs (10)**	23.5	83.1	18.8	10.4	4.1	16.3	-3.1	6.7	1.9

Supplementary Table 8. Breakdown of contributions to the overall free energies of de-lithiation of **6** to form **7** – **10** (kcal mol^{-1}) using M_9 clusters.

	ΔE_{BS1}	ΔH_{BS1}	ΔG_{BS1}	$\Delta G_{BS1/bnz}$	$\Delta G_{BS1/bnz+D3BJ}$	ΔE_{BS2}	$\Delta G_{BS2/bnz+D3BJ}$	$\Delta E_{ZORA/bnz+D3BJ)}$	$\Delta G_{ZORA/bnz+D3BJ}$
**Na (7)**	3.5	3.3	3.4	0.5	-15.0	7.0	-11.5	-7.1	-7.2
**K (8)**	-3.5	-3.8	-4.5	-5.8	-14.9	-12.7	-24.1	-19.5	-20.5
**Rb (9)**	1.1	0.9	1.2	-0.6	-9.8	-4.9	-15.8	-17.8	-17.7
**Cs (10)**	-3.9	56.9	-4.8	-6.5	-13.9	-10.1	-20.2	-19.7	-20.7

References

1. R. J. Schwamm, M. S. Hill, H. Y. Liu, M. F. Mahon, C. L. M[c]Mullin, and N. A. Rajabi, *Chem. Eur. J.*, 2021, **27**, 14971-14980.
2. K. G. Pearce, M. S. Hill and M. F. Mahon, *Chem. Commun.*, 2023, **59**, 1453-1456.
3. J. Hicks, M. Juckel, A. Paparo, D. Dange and C. Jones, *Organometallics*, 2018, **37**, 4810-4813.
4. O. V. Dolomanov, L. J. Bourhis, R. J. Gildea, J. A. K. Howard and H. J. Puschmann, *J. Appl. Cryst.* 2009, **42**, 339-341.
5. G. M. Sheldrick, *Acta Cryst.* 2015, **A71**, 3-8.
6. G. M. Sheldrick, *Acta Cryst.* 2015, **C71**, 3-8.
7. M. J. Frisch, G. W. Trucks, H. B. Schlegel, G. E. Scuseria, M. A. Robb, J. R. Cheeseman, G. Scalmani, V. Barone, G. A. Petersson, H. Nakatsuji, X. Li, M. Caricato, A. V. Marenich, J. Bloino, B. G. Janesko, R. Gomperts, B. Mennucci, H. P. Hratchian, J. V. Ortiz, A. F. Izmaylov, J. L. Sonnenberg, Williams, F. Ding, F. Lipparini, F. Egidi, J. Goings, B. Peng, A. Petrone, T. Henderson, D. Ranasinghe, V. G. Zakrzewski, J. Gao, N. Rega, G. Zheng, W. Liang, M. Hada, M. Ehara, K. Toyota, R. Fukuda, J. Hasegawa, M. Ishida, T. Nakajima, Y. Honda, O. Kitao, H. Nakai, T. Vreven, K. Throssell, J. A. Montgomery Jr., J. E. Peralta, F. Ogliaro, M. J. Bearpark, J. J. Heyd, E. N. Brothers, K. N. Kudin, V. N. Staroverov, T. A. Keith, R. Kobayashi, J. Normand, K. Raghavachari, A. P. Rendell, J. C. Burant, S. S. Iyengar, J. Tomasi, M. Cossi, J. M. Millam, M. Klene, C. Adamo, R. Cammi, J. W. Ochterski, R. L. Martin, K. Morokuma, O. Farkas, J. B. Foresman, D. J. Fox, Wallingford, CT, 2016.
8. D. Andrae, U. Häußermann, M. Dolg, H. Stoll, H. Preuß, *Theor. Chim. Acta* 1990, **77**, 123-141.
9. a) P. C. Hariharan, J. A. Pople, *Theor. Chim. Acta* 1973, **28**, 213-222; b) W. J. Hehre, R. Ditchfield, J. A. Pople, *J. Chem. Phys*. 1972, **56**, 2257-2261.
10. Höllwarth, A.; Böhme, M.; Dapprich, S.; Ehlers, A. W.; Gobbi, A.; Jonas, V.; Köhler, K. F.; Stegmann, R.; Veldkamp, A.; Frenking, G. *Chem. Phys. Lett.* 1993, **208**, 237.
11. a) A. D. Becke, *Phys. Rev. A* 1988, **38**, 3098-3100; b) J. P. Perdew, *Phys. Rev. B* 1986, **33**, 8822-8824.
12. J. Tomasi, B. Mennucci, R. Cammi, *Chem. Rev.* 2005, **105**, 2999-3094.
13. S. Grimme, S. Ehrlich, L. Goerigk, *J. Comp. Chem.* 2011, **32**, 1456-1465.
14. a) Leininger, T., Nicklass, A., Küchle, W., Stoll, H., Dolg, M., Bergner, A. The accuracy of the pseudopotential approximation: non-frozen-core effects for spectroscopic constants of alkali fluorides XF (X = K, Rb, Cs) *Chem. Phys. Lett*. 1996, **255**, 274-280; b) Weigend, F., Ahlrichs, R. Balanced basis sets of split valence, triple zeta valence and quadruple zeta valence quality for H to Rn: Design and assessment of accuracy *Phys. Chem. Chem. Phys*. 2005 **7**, 3297.
15. a) Noro, Takeshi, Sekiya, Masahiro, Koga, Toshikatsu Correlating basis sets for the H atom and the alkali-metal atoms from Li to Rb *Theor. Chem. Acc.* 2003, **109**, 85-90; b) Noro, Takeshi,

Sekiya, Masahiro, Koga, Toshikatsu Segmented contracted basis sets for atoms H through Xe: Sapporo-(DK)-nZP sets (n = D, T, Q) *Theoretical Chemistry Accounts* 2012, **131**, 1124; c) Yamamoto, Hironori, Matsuoka, Osamu Accurately Energy-Optimized Gaussian Basis Sets for Hydrogen 1s through 5g Orbitals *Bull. Univ. Electro. Comm.* 1992, **5**, 23-34 (1992) In Japanese. Citation at https://ci.nii.ac.jp/naid/40004737908/en/.

16. https://wires.onlinelibrary.wiley.com/doi/full/10.1002/wcms.1606.
17. a) Pantazis, D. A.; Chen, X.-Y.; Landis, C. R.; Neese, F. *J. Chem. Theory Comput*. 2008, **4**, 908; b) Buhl, M.; Reimann, C.; Pantazis, D. A.; Bredow, T.; Neese, F. *J. Chem. Theory Comput.* 2008, **4**, 1449; c) Pantazis, D. A.; Neese, F. *J. Chem. Theory Comput.*, 2009, **5**, 2229; d) Pantazis, D. A.; Neese, F. *J. Chem. Theory Comput.* 2011, **7**, 677; e) Pantazis, D. A.; Neese, F. *Theor. Chem. Acc.*, 2012, **131**, 1292; f) Rolfes, J. D.; Neese, F.; Pantazis, D. A. *J. Comput. Chem.* 2020, **41**, 1842.
18. a) van Lenthe, E.; Baerends, E. J.; Snijders, J. G. *J. Chem. Phys.*, 1993, **99**(6), 4597; b) C. van Wuellen, *J. Chem. Phys.* 1998, **109**, 392-399.
19. Exp and comp (Armentrout) – values for BDEs of M^+ to benzene, https://pubs.acs.org/doi/epdf/10.1021/jp002652f.
20. Cox, J. D., Wagman, D. D., and Medvedev, V. A., CODATA Key Values for Thermodynamics, Hemisphere Publishing Corp., New York, 1989.
